# Protective potential of selected microbial and non-microbial biostimulants against *Zymoseptoria tritici* leaf blotch in winter wheat as affected by the form of N supply

**DOI:** 10.3389/fpls.2024.1407585

**Published:** 2024-09-27

**Authors:** Markus Göbel, Samiksha Dulal, Lea Sommer, Markus Weinmann, Abdullah Al Mamun, Aneesh Ahmed, Neerakkal Sujeeth, Karin Mai, Günter Neumann, Torsten Müller, Klára Bradáčová

**Affiliations:** ^1^ Institute of Crop Science, Fertilization and Soil Matter Dynamics, University of Hohenheim, Stuttgart, Germany; ^2^ Institute of Crop Science, Nutritional Crop Physiology, University of Hohenheim, Stuttgart, Germany; ^3^ BioAtlantis Ltd., Clash Industrial Estate, Tralee, County Kerry, Ireland; ^4^ SP Sourcon Padena GmbH, Research and Development, Tübingen, Germany

**Keywords:** biotic stress, *Zymoseptoria tritici*, winter wheat, seaweed extracts, microbial consortium, agriculture, nitrate, ammonium

## Abstract

**Introduction:**

The production of high-quality food for the growing world population on the one hand and the reduction of chemical-synthetic pesticides on the other hand represents a major challenge for agriculture worldwide. The effectiveness of a combination of microbial and non-microbial biostimulants (BSs) with various nitrogen (N) forms in pathogen defense is discussed as a promising, but still poorly understood bio-based alternative for crop protection.

**Methods:**

For this reason, nitrate and stabilized ammonium fertilizer both combined with a consortium of *Pseudomonas brassicacearum*, *Bacillus amyloliquefaciens*, and *Trichoderma harzianum* as soil treatment or with a mixture of seaweed extract (*Ascophyllum nodosum*) together with chitosan-amended micronutrient fertilizer as foliar spray application were compared under controlled greenhouse conditions. Furthermore, a combination of microbial and different non-microbial BSs (seaweed extracts + chitosan) and micronutrients with nitrate or with stabilized ammonium fertilizer was tested under field conditions to improve nutrient availability, promote plant growth, and suppress *Zymoseptoria tritici* (*Zt*) in winter wheat.

**Results and discussion:**

While plant-protective effects against *Zt* by the microbial consortium application could be observed particularly under ammonium fertilization, the application of seaweed extract–chitosan mixture expressed plant defense against *Zt* more strongly under nitrate fertilization. In the field trial, the combination of microbial consortium with the seaweed extract–chitosan mixture together with micronutrients zinc (Zn) and manganese (Mn) showed positive effects against *Zt* under ammonium fertilization, associated with increased levels of defense metabolites. Furthermore, the additional input of Zn and copper (Cu) from the chitosan application improved the micronutrient status by minimizing the risk of Zn and Cu deficiency under controlled and field conditions. The use of BSs and the inoculation of *Zt* did not show any effects on plant growth and yield neither under controlled greenhouse conditions nor in the field. Summarized, microbial and non-microbial BSs separately applied or even combined together as one treatment did not influence plant growth or yield but made a positive contribution to an N form-dependent promotion of pathogen defense.

## 1 Introduction

One of the most critical fungal pathogens in wheat is *Zymoseptoria tritici* (*Zt*) (Quaedvlieg et al., 2011), which leads to yield losses of 5%–10% and reduction of food quality worldwide (Fones and Gurr, 2015). Intensive farming with the application of pesticides against pathogens such as *Zt* and chemical fertilizers is often regarded as necessary to feed the world’s growing population and to guarantee food security, but at the same time, this industrialized style of agriculture has a negative impact on the environment. Many of those pesticides are assumed to be readily degraded and removed from the soil. It was later shown that they can form bound residues, formerly undetected, and are even found as pesticide residues in food (Pogacean and Gavrilescu, 2009; Raliya et al., 2018). This agricultural challenge points out the relevance of an improved and sustainable cropping system avoiding excessive use of chemical-synthetic pesticides for environmental protection and high-quality food supply (Zimmermann et al., 2021). Targeted mineral fertilization with the focus on nitrogen (N) is most common for high plant growth and crop yield, even though its pathogen-suppressing effect is not less relevant (Huber and Haneklaus, 2007) but has been neglected in the past. However, it has been confirmed that adequate mineral N fertilization can improve plant tolerance to biotic and abiotic stresses (Fernandes and Rossiello, 1995; Walters and Bingham, 2007; Geisseler and Scow, 2014; Ding et al., 2021). Other studies proved the dependence of the effect of N fertilization on plant tolerance to the disease susceptibility of the crop varieties (Huber and Watson, 1974) or the importance of the source of N and the rate of application (Baker and Martinson, 1970; Huber and Watson, 1974). On the one hand, the impact of nitrate fertilization on plant tolerance correlates with the applied amount. The higher the N application is, the higher the susceptibility to certain diseases. Nevertheless, it has to be mentioned that the effect of nitrogen depends on the biology of the pathogen and the response of the crop plant. For example, a dense canopy formation can create a microclimate favorable for fungal infections. Furthermore, the nitrogen effect depends on the difference between biotrophic or saprophytic fungi and the influence of the nitrogen form (Weinmann et al., 2023). On the other hand, Sun et al. (2020) mentioned in their research that both ammonium and nitrate fertilization can promote or suppress plant diseases equally. This may depend on the type of pathogen and whether the ammonium fertilizer was stabilized or not, because of fast nitrification in aerated soils ammonium fertilizer show almost the same effect as nitrate. To combine the different N forms with microbial biostimulants (BSs), a consortium of plant-growth-promoting microorganisms (PGPMs) is supposed to be more effective than single PGPMs to strengthen plants against biotic and abiotic stresses (Mamun et al., 2024). Furthermore, a microbial consortium offers a broader range of usage than single strains due to the versatile compounds which are present in the consortium (Sarma et al., 2015; Bradáčová et al., 2019). Bradáčová et al. (2020) and Mamun et al. (2024) additionally identified that the form of N supply can be decisive for the efficiency of microbial BSs. Stabilized ammonium nutrition in combination with microbial consortium of *Pseudomonas* spp., *Bacillus* spp., and *Trichoderma* spp. improved the uptake of ammonium-N and led to increased phosphorus (P) concentrations in maize shoot tissues compared to nitrate supply. Increased root length was also observed with stabilized ammonium supply compared to nitrate supply. In addition, Halpern et al. (2015) recorded many positive abilities of a diversity of microorganisms, such as increased root growth due to the production of phytohormones, higher phosphate availability through mineralization of organic P, solubilization of iron (Fe) through chelating siderophores produced by the bacteria, and enhanced accumulation of Zn and potassium (K) in the plant tissue by excreting organic acids resulting in higher plant uptake and yield. Non-microbial BSs such as chitin, chitosan, or seaweed extracts also perform more efficiently as combined treatments to improve disease tolerance according to several studies (Nanda et al., 2021). Apart from stress priming effects, seaweed extracts in higher concentrations can contribute to enhanced solubility of micronutrients chelating them by large organic molecules and affect the nutrient uptake (Halpern et al., 2015). Furthermore, it is reported that chitosan can reduce the water content in cells due to its hydrophilic nature to mitigate stress and increases root length by acting as an additional source of carbon, resulting in improved nutrient uptake (Shahrajabian et al., 2021). Regarding the application of the micronutrients Zn and Mn, a Mn-dependent superoxide dismutase (MnSOD) is produced as a defense reaction by plants due to biotic and abiotic stresses (Kumar et al., 2020). Furthermore, Mn is involved in the synthesis of toxic phenols and lignification by forming a physical barrier against fungal pathogens. Similar to Mn, Zn acts as a cofactor for enzymes such as superoxide dismutase, and can increase the production of total phenolics or total antioxidants resulting in protective plant responses (Silva et al., 2022). In this multicomponent approach, the effect of microbial (*Pseudomonas brassicacearum*, *Bacillus amyloliquefaciens*, and *Trichoderma harzianum*) and non-microbial (seaweed extracts + chitosan) BSs and micronutrients (Zn and Mn) on *Zt* affected winter wheat with different N supplies was investigated. To examine both, the plant growth promoting potential and the plant protecting potential of biostimulants (BSs) combined with ammonium or nitrate fertilization, nutrient analyses, and metabolic assessments were performed. As complex treatments were tested in this study, it is hypothesized that beneficial effects on plant performance, nutrient acquisition, and tolerance of wheat plants result from a complexity of interactions of these treatments with the plant and its environment. However, the experimental setups do not allow for an isolation of effects that could be clearly attributed to certain components of the complex treatments.

We hypothesized that:

1. the application of different N forms together with a microbial consortium or seaweed extract in combination with chitosan improves plant growth and development by enhanced root growth and nutrient uptake under biotic stress conditions in the greenhouse.
2. the combination of stabilized ammonium fertilizers with microbial BSs, non-microbial BSs, and micronutrients leads to increased plant growth and enhanced disease suppression of *Zt*-inoculated wheat plants in the field.
3. different N forms combined with various BSs alleviate the effects of *Zt* infestation by inducing increased production of defense metabolites under greenhouse and field conditions.

The overall objective of this study was to examine the effect of different N forms in combination with different BSs on the suppression of *Zt*.

### 1.1 Pot experiment

#### 1.1.1 Experimental setup

Winter wheat *Triticum aestivum* L. Asory (SECOBRA Saatzucht GmbH, Unterschleißheim, Germany), a medium-late ripening variety and showing a moderately tolerance to *Zt*, was cultivated from 05/05/2022 to 29/06/2022 (55 days) in a greenhouse at a mean temperature of 28.7°C and a mean relative humidity of 45.7%. Plastic cylinders with a diameter of 95 mm (pot surface 71 cm^2^), and a height of 210 mm were used as pots. Each pot contained 1,173 g soil (Filderlehm 2015) from the experimental station Heidfeldhof of the University of Hohenheim) and 587 g quartz sand mixture (0.6 – 1.2 mm) (66.5:33.5 w:w). An overview on physical and chemical soil properties is shown in **Supplementary Table 1**. Per pot, 12 wheat seeds were sown. A 100-g layer of washed quartz sand (0.6 – 1.2 mm) was added on top of the soil surface to reduce evaporation and pest pressure and avoid siltation by irrigation. Each pot was fertilized with 120 mg P kg^−1^ DW in the form of Ca(H_2_PO_4_)_2_, 150 mg K kg^−1^ DW in the form of K_2_SO_4_, and 50 mg Mg kg^−1^ DW in the form of MgSO_4_ and sprayed on the soil–sand mixture while the substrate was mixed by hand before filling into the pots. The two different nitrogen treatments, calcium nitrate (YaraTera^®^ CALCINIT^®^, YARA GmbH & Co. KG, Dülmen, Germany) and ammonium sulfate (NovaTec^®^ Solub 21, COMPO EXPERT GmbH, Münster, Germany) with a nitrification inhibitor [3,4-dimethyl-1H-pyrazole phosphate (DMPP)], were fertilized each with 100 mg kg^−1^ DW as milled powder mixed with the soil–sand mixture before filling into the pots. The pots were regularly watered by weight up to 70% of the water holding capacity (WHC) when the water content in the soil/sand substrate fell below 50% of the WHC. The pots were arranged according to a split-plot design with two real repetitions and two main plots per repetition (**Supplementary Figure 1**). In total, 50 pots were prepared, 10 different variants with five repetitions per treatment (**Supplementary Table 2**). Within the main plots, treatments were arranged on two tables (table one with two replicates and table two with three replicates) as randomized complete block design (RCBD). The model for this design was


$$y_{ijkl}=\;\mu \;+\;t_{i}+\;r_{ij}+\tau_{k}+\;gt_{li}+\;e_{ijkl}$$

with *μ* as the overall effect, *t_i_* as the fixed/random effect of the *i*th table, *r_ij_* as the fixed effect of the *j*th block on the *i*th table, *τ_k_* as the effect of the *k*th treatment, *gt_li_* as the random effect of the *t*th main plot in the *j*th block on the *i*th table, and *e_ijkl_* as the error of *y_ijkl_*.

#### 1.1.2 Application of biostimulants

As biostimulant products, a microbial consortium composed of different microorganisms and a mixture of seaweed extract with chitosan was tested. The consortium (*Pseudomonas brassicacearum*: 2 × 10^10^ cfu g^−1^, *Bacillus amyloliquefaciens*: 2 × 10^10^ cfu g^−1^, and *Trichoderma harzianum*: 1 × 10^8^ cfu g^−1^, SP Sourcon Padena GmbH, Tübingen, Germany) formulated with milk powder that served as a carrier and nutrient medium was applied as a soil application on the sowing day shortly before sowing. The 10-g package of consortium powder (for the 10^7^ cm^2^ soil surface) was dissolved into 500 ml of distilled water. From the stock solution (SL), 35.5 µl (0.05 µl cm^−2^) were pipetted and mixed in a beaker with 100 ml of distilled water using a magnetic stirrer. For each pot, 10 ml SL (0.14 ml cm^−2^) was dropped onto the top layer of the soil and then carefully mixed into the soil by hand. The seaweed extract (*Ascophyllum nodosum* extract, BioAtlantis Ltd., Tralee, Ireland) together with chitosan as a micronutrient formulation with surfactants containing the mixture of micronutrients together with copper (Cu), manganese (Mn), molybdenum (Mo), and zinc (Zn); 7.1 N (0.55 NH_4_-N+6.55 NH_2_N) + 11.8 K + 3.4 S + <0.1 Cl + 1.42 Cu + 2.84 Mn + 2.23 Zn + 0.028 Mo w/v [g 100 ml^−1^ chitosan formulation] (Wuxal^®^ Micromix plus Chitosan; A7116, AGLUKON Spezialdünger GmbH & Co. KG, Düsseldorf, Germany), were applied as foliar spraying. The total amounts of mineral nutrients applied to the plants via chitosan are shown in **Supplementary Table 3**. In accordance with the BioAtlantis’ application recommendation, the seaweed + chitosan mixture was applied 2 days prior to a biotic stress event and every 5 days thereafter if the disease intensity was >25% of infested leaf area or every 7 days thereafter if the disease spread was <25% of infested leaf area with 2 l seaweed extract ha^−1^ and 2 l chitosan ha^−1^ (0.02 µl cm^−2^) in 250 l water ha^−1^ (2.5 µl water cm^−2^). In total, five applications have been performed during the pot experiment. An SL was prepared as follows: 15.62 µl of the seaweed extract was pipetted in one 2 ml Eppendorf tube and the same amount of chitosan in another 2 ml Eppendorf tube, both filled up with distilled water to 1 ml. Both tubes were properly mixed by use of an orbital shaker, then 968 µl of distilled water was added in each and stirred again. Finally, the contents of both Eppendorf tubes were combined with 200 ml of distilled water in a glass beaker and mixed with a magnetic stirrer. Of this application mixture, 18.2 ml was sprayed onto the leaves of the plants of each pot.

#### 1.1.3 *Zymoseptoria tritici* cultivation, inoculation, and disease assessment

YMDA–agar YMB–liquid culture medium as the most efficient culture media for inoculum production according to Saidi et al. (2012) was selected for the *Zt* cultivation. 4 g Bacto™ Yeast Extract Technical (A288620; Becton, Dickinson and Company, Franklin Lakes, United States) 4 g Bacto™ Malt Extract (A218630; Becton, Dickinson and Company, Franklin Lakes, United States), 10 g (D(+)-glucose 1-hydrate), (A143140.1211; AppliChem GmbH, Darmstadt, Germany), and 15 g Bacter Agar (A0949, 1000; AppliChem GmbH, Darmstadt, Germany) were dissolved in 1 l of distilled water. The solution was mixed properly with a magnetic stirrer and autoclaved (121°C, 20 min). Afterwards, the solution was poured into sterile Petri dishes and solidified during cooling. With a flame-sterilized spatula, a piece of *Zt* fungal mycelium was gouged out and placed *vice versa* on the agar-medium, and the Petri dishes were locked with parafilm and stored in a light cabinet for 1–3 weeks at room temperature (18°C–20°C), with the culture medium facing the above. The YMB–liquid culture medium was prepared, consisting of 4 g YE (Bacto™ Yeast Extract Technical, A288620; Becton, Dickinson and Company, Franklin Lakes, United States), 4 g ME (Bacto™ Malt Extract, A218630; Becton, Dickinson and Company, Franklin Lakes, United States), and 10 g glucose (D(+)-glucose 1-hydrate) (A143140.1211; AppliChem GmbH, Darmstadt, Germany) filled up to 1 l with distilled water. The solution was mixed with a magnetic stirrer and autoclaved (121°C, 20 min). In a 1 l Erlenmeyer flask, two complete *Zt* plates were mixed with 500 ml of liquid YMB medium. In a 0.5 l Erlenmeyer flask, one complete *Zt* plate was mixed with 333 ml liquid YMB medium. The *Zt* fungi on the plates were gouged out with a flame-sterilized spatula and cut into pieces before adding them to the liquid medium. Everything together was mixed in a sterile cabinet and put on a shaker with a frequency of 100 - 125 rounds per minute (rpm) for 2 weeks at 18°C–20°C. Thereafter, the YMB medium was poured through an autoclaved, two-layered gauze bandages into an autoclaved vessel. The residue represented the mycelium, and the filtrate contained the *Zt* spores. The filtrate was centrifuged at 20°C for 10 min and 6,000 min^-1^ (17,307×*g*) (Sorvall RC 6 Plus centrifuge, Thermo Electron LED GmbH, Osterode, Germany), and the supernatant was discarded. The remaining *Zt* spores were then diluted with autoclaved water, filled into tubes, and mixed with an orbital shaker. The spore suspension was filled from the tubes into 0.5 l PET bottles which were stored at −40°C until they were used. The *Zt* spores were counted with a “Fuchs-Rosenthal” cell chamber. Three 2 ml Eppendorf tubes with a 1:1,000 concentration of *Zt* spores were used, and 3.2 µl was pipetted in the counting chamber. There were 16 group squares existing in the chamber; one group square included 16 small squares. For every sample, five group squares were counted and the mean value was determined. Lastly, the mean value from all three counts was 374.8 × 10^7^ spores ml^−1^. *Zt* was applied with a small sprayer and a concentration of 1 × 10^7^ spores ml^−1^ *Zt* suspension 15 days after sowing (DAS). 355 μl *Zt* suspension/pot (5 μl cm^−2^ soil surface) was applied. The pots were covered for 3 days with four different foil tunnels with wet towels inside to keep a high relative humidity. For keeping the humidity even higher, a transparent plastic foil was additionally placed over the tunnels. Visual assessment of *Zt* infestation was done 29, 34, 43, 49, and 55 DAS. The percentage of leaf area covered with *Zt* for six youngest, fully developed leaves from six different plants per pot were evaluated based on the method of James W.C. described by Eyal et al. (1987).

#### 1.1.4 Plant analyses

The dry weight of the aboveground plant tissue and root samples per pot was determined after harvest at 55 DAS. Aliquots of washed root samples were stored in 70% (v/v) ethanol and analyzed for root length and morphological structure (i.e., root diameter classes) with an Epson Expression 10000XL scanner (Seiko Epson K.K., Japan) using WinRHIZO root analysis software package (Regent Instruments Inc., Quebec, Canada) (Moradtalab et al., 2020). For estimating the shoot and root dry weights, the plant tissue and root samples packed in paper bags were oven-dried at 60°C for 4 days and then weighed.

#### 1.1.5 Determination of stress metabolites

Fresh leaf samples from 27 DAS and 55 DAS were used for the determination of selected physiological stress indicators such as hydrogen peroxide (H_2_O_2_), ascorbate peroxidase (APX; EC 1.11.1.11) activity, and guaiacol peroxidase (GPX; EC 1.11.1.7) activity after homogenization of 0.1 g of plant tissues shock-frozen in liquid nitrogen in 1.5 ml of 50 mM potassium phosphate extraction buffer, followed by centrifugation for 10 min at 14,000 min^−1^ (20,160×*g*) (Hettich centrifuge MIKRO 24-48 R, Tuttlingen, Germany). APX activity was recorded spectrophotometrically at 290 nm according to the method of Boominathan and Doran (2002). H_2_O_2_ levels were determined spectrophotometrically at 390 nm according to the method described by Moradtalab et al. (2018). GPX activity was performed spectrophotometrically at 470 nm using the tetra-guaiacol assay described by Moradtalab et al. (2020). For the determination of total antioxidants, leaf samples from 27 DAS were shock-frozen and homogenized in liquid nitrogen to use 0.1 g of fresh matter for methanolic extraction (80% v/v methanol) followed by centrifugation for 10 min at 14,000 min^−1^ (20,160×*g*) (Hettich MIKRO 24-48 R centrifuge, Tuttlingen, Germany). The 1,1-diphenyl-2-picrylhydrazyl radical (DPPH)-modified method was used to evaluate the free radical scavenging activity of antioxidants in the plant tissue (Moradtalab et al., 2020). A U-3300 spectrophotometer (Hitachi, Tokyo, Japan) was used for all spectrophotometric measurements.

#### 1.1.6 Analysis of mineral nutrients

The concentrations of the essential nutrients N, P, K, Ca, Mg, S, Zn, Mn, Fe, and Cu were determined in the oven-dried and milled plant tissues of final harvest (55 DAS). The shoots were ground in a disc-oscillating agate stone mill (SIEBTECHNIK GmbH, Mülheim-Ruhr, Germany) for 3 min – 4 min to a fine powder. 0.2 g of powdered plant tissue per sample was weighed into a quartz glass beaker, and 1 ml ultrapure water and 2.5 ml nitric acid (ROTIPURAN^®^ ≥65%, p.a., ISO; Carl Roth GmbH & Co. KG) were added. After microwave (“ultraCLAVE III”; Fa. MLS Leutkirch) digestion was accomplished, elemental concentrations were measured by inductive coupled plasma optical emission spectrometry (ICP-OES; “Agilent 5110”, Santa Clara, United States). Total nitrogen, carbon, and sulfur were determined with the Vario MAX CNS elemental analyzer (Elementar Analysensysteme GmbH, Langenselbold, Germany) (VDLUFA, 2012).

#### 1.1.7 Statistical evaluation

Statistical analyses were performed using SAS/STAT software package of SAS^®^ 9.4 (2016) (SAS Institute Inc., Cary, USA). A one-way ANOVA followed by a Tukey test (*p* < 0.05 significance level) was used to compare means for statistically significant differences. Data are presented as mean values. Normal distributions and variance homogeneities of the residuals were checked by the Shapiro–Wilk test and by Levene’s test, respectively, and graphically against the predicted values by QQ plots, histograms, and graphs of the residuals according to Kozak and Piepho (2017).

### 1.2 Field experiment

#### 1.2.1 Experimental setup

Similar to the pot experiment, winter wheat of the variety Asory (SECOBRA Saatzucht GmbH, Unterschleißheim, Germany) was the test plant in the field experiment. This variety is described as moderately tolerant to *Zt* with medium plant length and standability, and medium-late ripening quality. It was cultivated from 20/10/2021 to 27/07/2022 (280 days) with a sowing density of 330 grains m^-2^ at the experimental station Heidfeldhof of the University of Hohenheim, Filderhauptstraße 201, 70599 Stuttgart, Germany (48°42′56.21 N, 9°11′15.64 E, 402 m above sea level, mean temperature of 8.8°C, mean relative humidity of 76.6%). A 3.28 ha field was used for the experiment with a plot size of 6 m × 8 m (48 m^2^). The sowing was performed with Amazone AD 303 (Amazonen-Werke H. Dreyer SE & Co. KG, Hasbergen, Germany) with a 3 m working width and front cultivator, a 3.5 cm sowing depth, and a 12.5 cm row spacing. The soil texture in the field was loamy clay, and the soil type was Cambisol. The field was tilled with a rotary harrow twice before sowing. Five days after sowing and during field emergence (BBCH 23), weeding/hoeing was undertaken to control the weeds, except for the negative control variant 27. Soil sampling for N_min_ measurements was performed during the vegetation period for the whole field for fertilizer requirement calculation on 03/03/2022 (N_min_ = 38.46 kg N ha^−1^). Furthermore, after harvesting, plot-specific sampling for N_min_ analyses was performed on 11/08/2022. One sampling was performed to collect soil samples from 0–30-cm, 30–60-cm, and 60–90-cm depths according to VDLUFA (2023). The results of the soil analyses are shown in **Supplementary Table 4**. The fertilizer "P, K, Mg plus S – fertilizer" containig 4.4 P +12.5 K + 3 Mg + 12 S (w/w) [kg 100 kg^−1^] (Beiselen GmbH, Ulm, Germany) was applied, broadcast twice: once 380 kg fertilizer ha^−1^ before tillage in September 2021 and 324 kg fertilizer ha^−1^ at the beginning of the vegetation (BBCH 22) on 03/03/2022 with Rauch Aero 1110 pneumatic spreader (Rauch Landmaschinenfabrik GmbH, Rheinmünster, Germany). In total, 31 kg P ha^−1^, 88 kg K ha^−1^, 21 kg Mg ha^−1^, and 84 kg S ha^−1^ were fertilized. The two different nitrogen treatments, calcium nitrate (YaraTera^®^ CALCINIT^®^, YARA GmbH & Co. KG, Dülmen, Germany) and ammonium sulfate (NovaTec^®^ Solub 21, COMPO EXPERT GmbH, Münster, Germany) with nitrification inhibitor [3,4-dimethyl-1H- pyrazole phosphate (DMPP)], were fertilized each with application rates of 240 kg N ha^−1^ also with the Rauch Aero 1110 pneumatic spreader. The application of calcium nitrate was divided into three applications according to farming practice. The first one with 40% of the total amount of calcium nitrate (80.6 kg N ha^−1^) at BBCH 23 on 10/03/2022, the second one with 43% (86.7 kg N ha^−1^) at BBCH 30 on 22/04/2022, and the third one with 17% (34.3 kg N ha^−1^) at BBCH 33–37 on 20/05/2022.

The application of ammonium sulfate was divided into two applications: the first one with 60% of the total amount of ammonium sulfate (120.9 kg N ha^−1^) at BBCH 23 on 10/03/2022, and the second one with 40% (80.6 kg N ha^−1^) at BBCH 33–37 on 20/05/2022. The herbicide Artus® (Cheminova Deutschland GmbH & Co. KG, Stade, Germany) was sprayed with 50 g ha^−1^ at BBCH 23; the herbicides Biathlon® 4D + Dash® E.C. (70 g ha^-1^ + 1 l ha^-1^; BASF SE, Limburgerhof, Germany) were sprayed together at BBCH 30. The herbicides were mixed with 300 l of water using an Amazone UF 901 (Amazonen-Werke H. Dreyer SE & Co. KG, Hasbergen, Germany) field sprayer (nozzle type IDKN 120-03, 3–4 bar, application volume 300 l ha^−1^, driving speed 5 km/h; Lechler GmbH, Metzingen, Germany). Variants treated with herbicides were also treated with the calcium nitrate fertilizer and therefore performed as a positive control treatment. The experimental design was a row-column design latinized in columns and blocks (Richter et al., 2009) (**Supplementary Figure 2**). There were 28 different variants with four repetitions for each sharing 112 plots in total (**Supplementary Table 5**). Ten variants were investigated in this study.

#### 1.2.2 Application of biostimulants

As BS products, a combined treatment of seaweed extract + chitosan mixture, micronutrients Zn and Mn, and a milk powder as a carrier medium for the microbial consortium of different microorganisms was used. The consortium (*Pseudomonas brassicacearum*: 2 × 10^10^ cfu g^−1^, *Bacillus amyloliquefaciens*: 2 × 10^10^ cfu g^−1^, and *Trichoderma harzianum*: 1 × 10^8^ cfu g^−1^, SP Sourcon Padena GmbH, Tübingen, Germany) formulated with milk was applied as a soil application on the sowing day shortly before sowing. A 5 g package of pure milk powder (for 500 m^2^) as a blank control and a 14 g package of milk powder mixed with the consortium (for 1,400 m^2^) were each dissolved into 500 ml of distilled water and filled up to 20 l of water to prepare the SL. The quantities applied were 1,920 ml SL milk powder plot^-1^ and 685.7 ml SL milk powder + consortium plot^-1^ each with three watering cans/plot. Each watering can was filled with 640 ml SL milk powder or 228.6 ml SL milk powder + consortium both along with 10 l of water applied simultaneously per plot in the longitudinal direction at first, and then perpendicular to the first application in the transverse direction of the plot, to ensure an even distribution of the products. The seaweed extract (*Ascophyllum nodosum* extract, BioAtlantis Ltd., Tralee, Ireland) together with WUXAL^®^ MICROMIX plus Chitosan (AGLUKON Spezialdünger GmbH & Co. KG, Düsseldorf, Germany) were applied as foliar application with an Amazone UF 901 (Amazonen-Werke H. Dreyer SE & Co. KG, Hasbergen, Germany) field sprayer (nozzle type IDKN 120-03, 3–4 bar, application volume 300 l ha^−1^, driving speed 5 km/h; Lechler GmbH, Metzingen, Germany). The total amounts of mineral nutrients applied to the plants via chitosan are shown in **Supplementary Table 6**. In accordance with AGLUKON’s application recommendation, the seaweed + chitosan mixture to be applied at shooting (BBCH 30) and at ear pushing (BBCH 51) was 2 l seaweed extract ha^−1^ and 2 l chitosan ha^−1^ (0.2 ml m^−2^) in 250 l water ha^-1^ (25 ml water m^-2^). An SL for 49 half plots was prepared as follows: 235.2 ml of seaweed extract and 235.2 ml of chitosan were filled up with distilled water to 1 l. The seaweed + chitosan solution got mixed with a magnetic stirrer and was filled in the tank of an Amazone UF 901 field sprayer with 29.4 l of water and 0.6 l seaweed + chitosan solution (25 ml m^−2^). Then, the SL was sprayed on the leaves of the plants per plot. Micronutrients Zn and Mn (Lebosol^®^-Zink^700^SC; 40% total Zn as zinc oxide 700 g Zn l^−1^; Lebosol^®^-Mangan^500^SC; 27.9% total Mn as manganese carbonate 500 g Mn l^−1^; Lebosol^®^ Dünger GmbH, Elmstein, Germany) were used for foliar application with an Amazone UF 901 field sprayer (nozzle type IDKN 120-03, 3–4 bar, application volume 300 l ha^−1^, driving speed 5 km/h; Lechler GmbH, Metzingen, Germany). In accordance with the manufacturer’s application recommendation, the Zn and Mn to be applied about 10 days after the start of vegetation, at BBCH 14 (3–4 days before *Zt* inoculation) were each 0.3 l Zn ha^−1^ and 0.5 l Mn ha^−1^ in 200 l of water (1.4 ml Zn plot^-1^ and 2.4 ml Mn plot^-1^ in 1 l of water), at BBCH 16 and at shooting/extension (BBCH 31) each 0.5 l Zn ha^−1^ and 0.75 l Mn ha^−1^ in 200 l of water (2.4 ml Zn plot^-1^ and 3.6 ml Mn plot^-1^ in 1 l of water).

#### 1.2.3 *Zymoseptoria tritici* cultivation, inoculation, and disease assessment

The process of *Zt* cultivation was performed as described in the pot experiment (see 1.1.3) based on Saidi et al. (2012). *Zt* was applied with a Hege 76 field sprayer (device carrier from Wintersteiger, Ried im Innkreis, Austria; field sprayer from Kubota, Osaka, Japan) at BBCH 23-24 (160 DAS) and a concentration of 1 × 10^7^ spores ml^−1^ *Zt* suspension. 100 ml *Zt* suspension m^−2^ (4.8 l plot^-1^) was applied. The *Zt* disease incidence was determined visually by evaluating the percentage of infested plants inside a 60 × 40 cm rectangle formed with a meter stick at BBCH 30-31 (190 DAS), BBCH 31-33 (204 DAS), and BBCH 75 (259 DAS) modified according to Moll et al. (2000).

#### 1.2.4 Plant analyses

The dry weight of five fully unfolded leaves from five different plants per plot at BBCH 33-37 (209 DAS) and three root samples per plot at BBCH 99 (278 DAS) were determined. To ensure the extraction of representative root samples, the shovel was placed carefully leaving sufficient space along the side and the puncture was created deep to the full height of the shovel blade (30 cm) to dig fresh roots out. The determination of the leaf and root dry weight as well as of the root length and morphological structure took place as in the pot experiment (see 1.1.4). Furthermore, for estimating grain nutritional analyses, grain samples (400 - 500 g per plot) from harvest at BBCH 99 (280 DAS) were oven-dried at 40°C for 4 days and then weighed.

#### 1.2.5 Determination of stress metabolites

Five fully unfolded leaves without visible disease symptoms from five different plants per plot from BBCH 75 (245 DAS) were used for the determination of selected physiological stress indicators with the methods as described in the pot experiment (see 1.1.5).

#### 1.2.6 Analysis of mineral nutrients

The concentrations of the essential nutrients N, P, K, Ca, Mg, S, Zn, Mn, Fe, and Cu were determined in oven-dried and milled leaf samples from BBCH 33-37 (209 DAS) and in 400 g–500 g oven-dried and milled grain samples per plot from harvest at BBCH 99 (280 DAS). Grinding of the leaf samples was the same process as for the pot experiment (see 1.1.6). 0.2 g of powdered leaf material and powdered grain material was weighed into a quartz glass beaker followed by the process of element analysis, which took place as described in the pot experiment (see 1.1.6).

#### 1.2.7 Statistical evaluation

Statistical analyses were performed using SAS/STAT software package of SAS^®^ 9.4 (2016) (SAS Institute Inc., Cary, USA). A one-way ANOVA followed by a Tukey test (*p* < 0.05 significance level) was used to compare means for statistically significant differences. Data are presented as mean values. Normal distributions and variance homogeneities of the residuals were checked by the Shapiro–Wilk test and by Levene’s test, as well as graphically against the predicted values by QQ plots, histograms, and graphs of the residuals according to Kozak and Piepho (2017).

## 2 Results

### 2.1 Pot experiment

#### 2.1.1 Disease severity affected by different N supplies and microbial and non-microbial biostimulants

**Figure 1:**
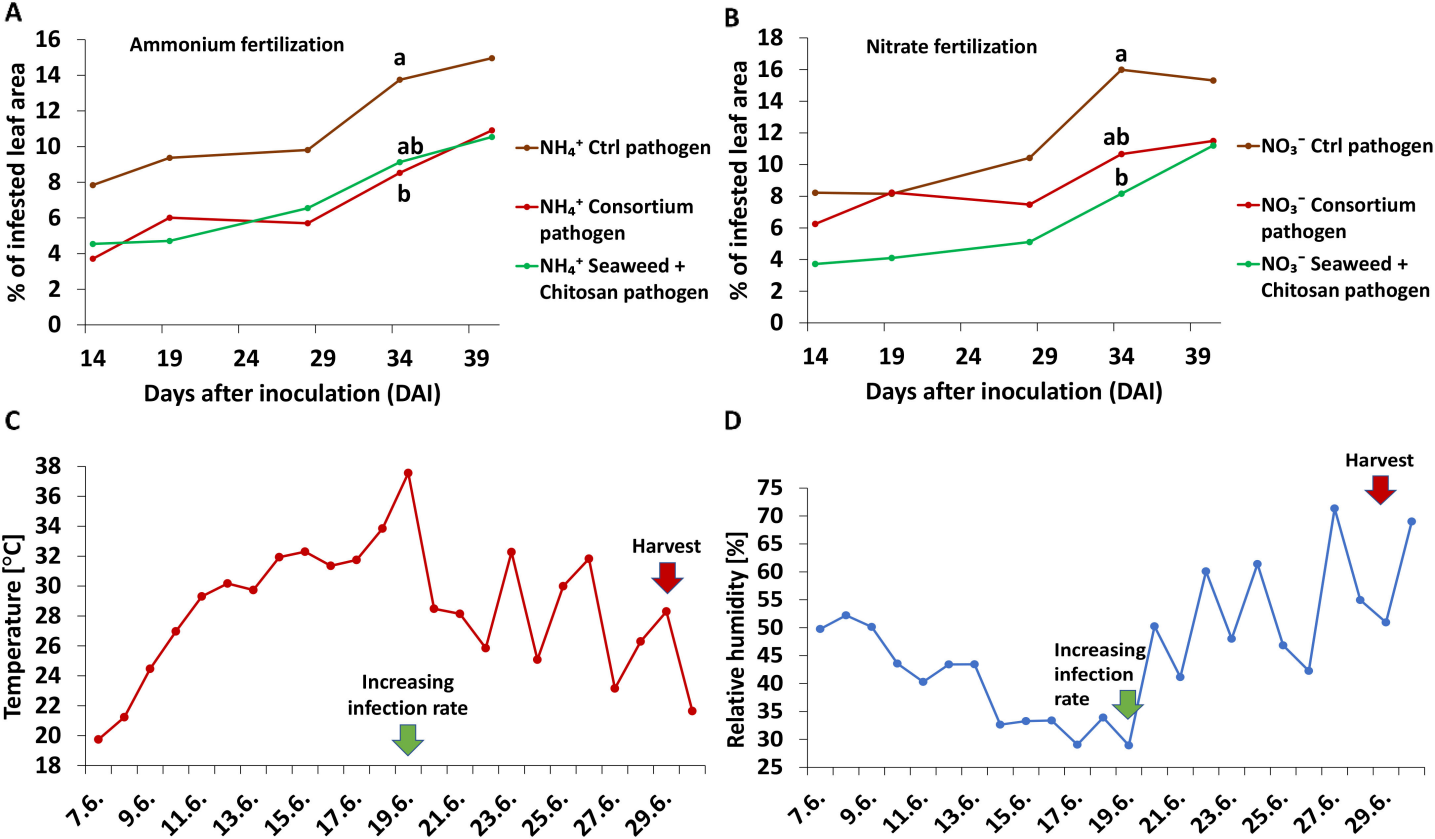
**(A, B)** Time course of disease spread of *Zymoseptoria tritici (Zt)* [% of infested leaf area] on winter wheat plants in the greenhouse treated with ammonium sulfate or calcium nitrate under control condition (brown lines) or with different biostimulants (microbial consortium, red lines; seaweed extract + chitosan, green lines) both inoculated with *Zt*. **(C)** Temperature course [°C] and **(D)** relative humidity course [%] over the plant growth period. Increasing infection rate from 19.06.2022 on. **(A, B)** represent mean values of five replicates per treatment. Mean values with at least one same or without lowercase letters within graph **(A, B)** are not significantly different according to Tukey test (α=0.05).

The *Zt* disease severity (DS) increased over time following a biphasic pattern with a slower relative increase of 25%–27% between 14 and 28 days after inoculation (DAI) and a steeper increase by 48%–53% over the next 12 days, finally reaching comparatively low absolute DS levels of 15% at the end of the culture period without N-form-dependent differences (**Figure 1**).

**Figure 2:**
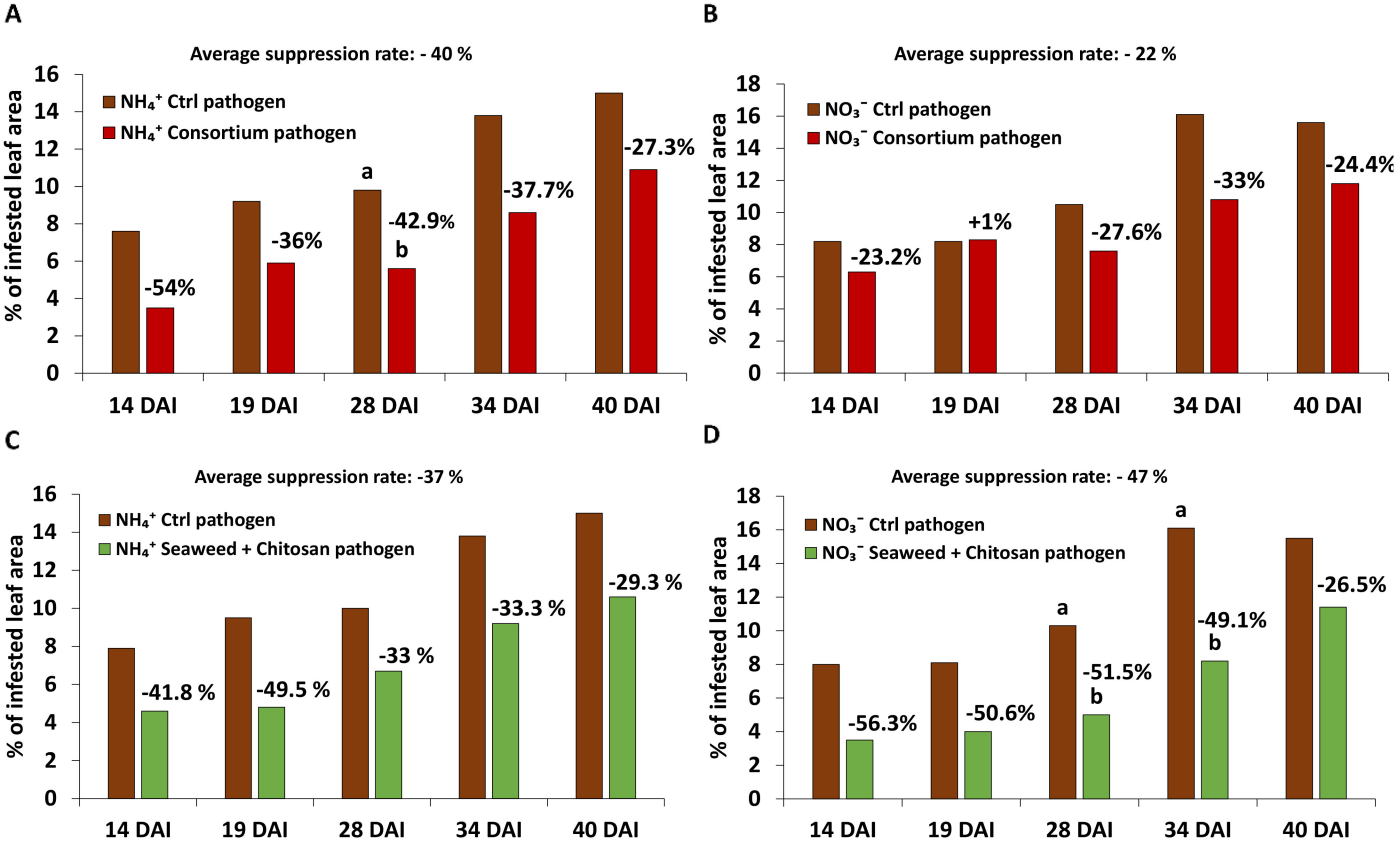
Disease severity [% of infested leaf area] of *Zymoseptoria tritici (Zt)* on winter wheat plants in the greenhouse 14, 19, 28, 34 and 40 days after inoculation (DAI) treated with **(A, C)** ammonium sulfate and **(B, D)** calcium nitrate, both under control condition (brown bars) and with different biostimulants (microbial consortium, red bars; seaweed extract + chitosan, green bars) inoculated with *Zt*. **(A–D)** represent mean values of five replicates per treatment. Mean values with the same or without lowercase letters within each graph are not significantly different according to Tukey test (α=0.05).

Protective effects of microbial consortium (MC) were particularly expressed under the ammonium nutrition [average DS suppression rate 40% (NH_4_^+^) vs. 22% (NO_3_^−^)] over the culture period. Furthermore, a clear trend although not significant at all time-points was detected (**Figures 2A, B**).

Protective effects of seaweed extract + chitosan (SC) appeared particularly under nitrate nutrition [average DS suppression rate 47% (NO_3_^−^) vs 37% (NH_4_^+^)]. Similar to MC, differences declined by the end of the culture period (**Figures 2C, D**).

#### 2.1.2 Defense metabolites

**Figure 3:**
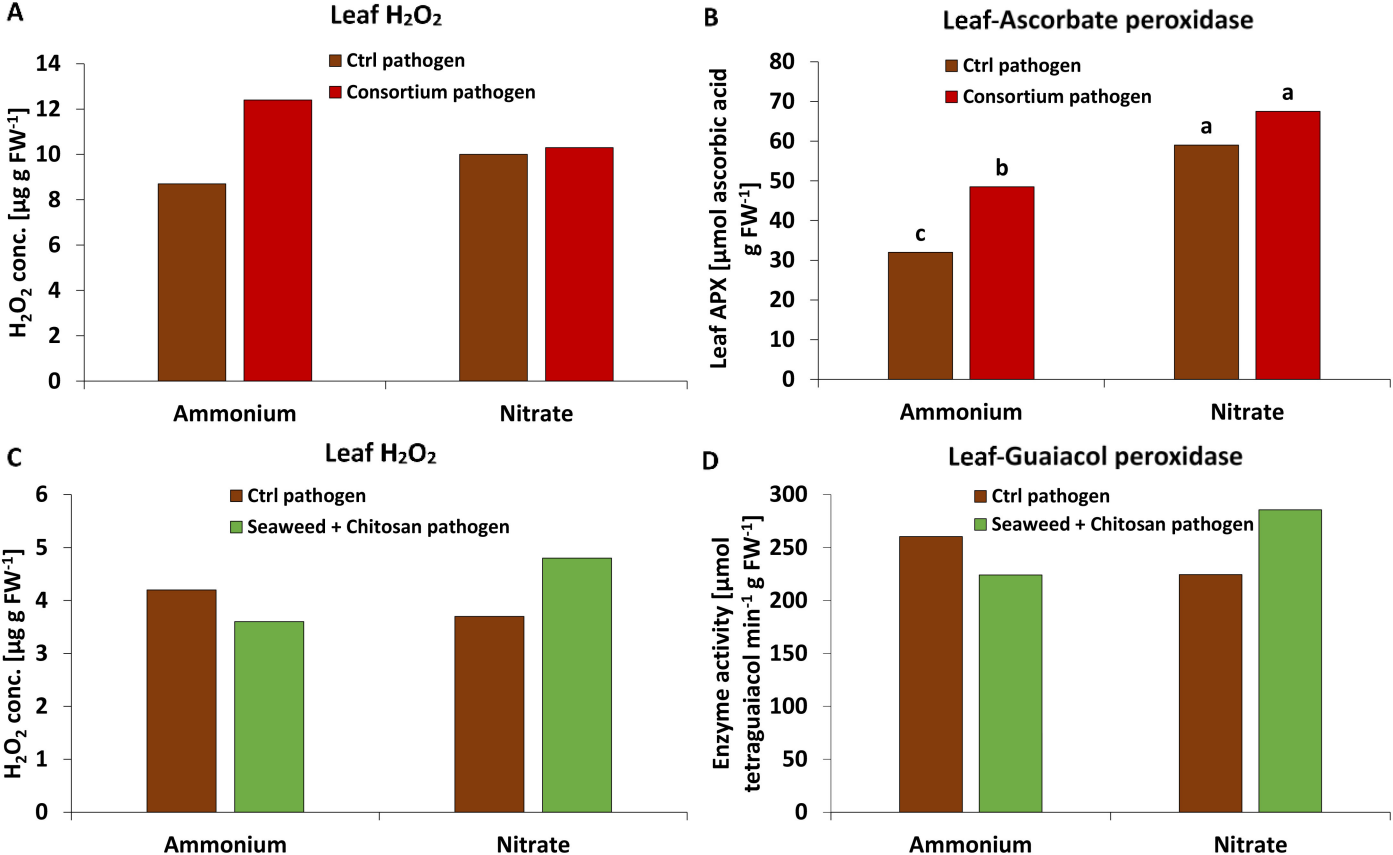
**(A, C)** Hydrogen peroxide (H_2_O_2_) concentration [ug g FW^-1^], **(B)** ascorbate peroxidase (APX) activity [μmol ascorbic acid g FW^-1^] and **(D)** guaiacol peroxidase (GPX) activity [μmol tetraguaiacol min^-1^ g FW^-1^] in the leaf tissue of winter wheat plants in the greenhouse treated with ammonium sulfate or calcium nitrate under control condition (brown bars) or with different biostimulants (microbial consortium, red bars (55 DAS); seaweed extract + chitosan, green bars (27 DAS)) both inoculated with *Zymoseptoria tritici (Zt)*. A represents mean values of three replicates in the ammonium control and ammonium- consortium treatments, five replicates in the nitrate control and nitrate-consortium treatments. **(B)** represents mean values of three replicates in the ammonium control and ammonium-consortium treatment, five replicates in the nitrate control and four replicates in the nitrate-consortium treatment. **(C, D)** represent mean values of five replicates per treatment. Mean values with the same or without lowercase letters within each graph are not significantly different according to Tukey test (α=0.05).

MC: At final harvest (55 DAS, 40 DAI), a trend of increased leaf H_2_O_2_ concentrations by 47% (**Figure 3A**) was associated with a similar but significant increase in APX activity (47%, **Figure 3B**) mediating H_2_O_2_ detoxification in MC-inoculated plants with NH_4_^+^ fertilization. No stimulatory effects of MC inoculation on H_2_O_2_ accumulation and APX activity were recorded under NO_3_^−^ supply (**Figures 3A, B**), but APX activity was generally significantly increased under NO_3_^−^ supply compared with plants supplied with NH_4_^+^ fertilization (**Figure 3B**). No comparable effects were detected at earlier developmental stages (27 DAS, 12 DAI), for GPX activity or for total antioxidants mediating non-enzymatic detoxification of reactive oxygen species (ROS) (**Supplementary Figure 3**).

SC: In contrast to MC-inoculated plants, earlier changes in defense metabolites were detectable already at 27 DAS (12 DAI). In plants with NO_3_^−^ fertilization, H_2_O_2_ accumulation in the leaf tissue tended to increase by 30% (**Figure 3C**). This was associated with an increase (27%) in the activity of GPX (**Figure 3D**). No comparable effects were detectable under NH_4_^+^ fertilization (**Figures 3C, D**), at later developmental stages (55 DAS, 40 DAI) or for APX activity. Also, no significant differences were detectable for total antioxidants (**Supplementary Figure 4**).

#### 2.1.3 Mineral nutritional status

For all treatments, the nutritional status of N, P, S, Mg, Fe, and Mn at final harvest was sufficient for adequate growth of wheat plants. Critical nutrient concentrations close or even below the reported deficiency threshold values (Bergmann, 1992) were recorded for K, Ca, Zn, and Cu. N form effects were detected for P, S, and Zn with increased leaf concentrations under NH_4_^+^ fertilization and for Ca, Mg, K, and Mn concentrations promoted under NO_3_^−^ supply (**Supplementary Table 7**).

**Figure 4:**
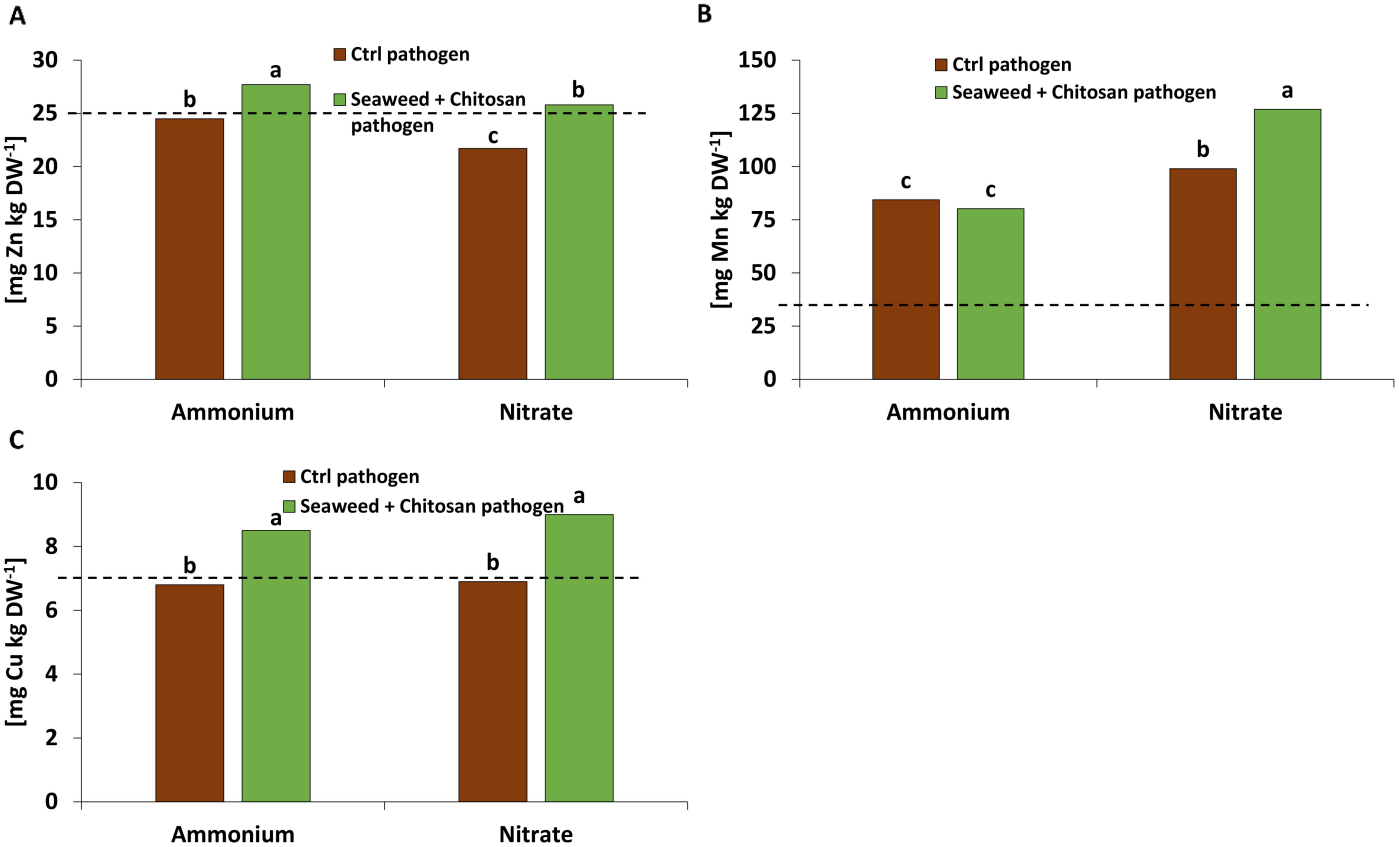
**(A)** Zinc (Zn), **(B)** manganese (Mn), **(C)** copper (Cu) concentration [mg kg DW^-1^] in the shoot tissue of winter wheat plants in the greenhouse 55 days after sowing (DAS) treated with ammonium sulfate or calcium nitrate under control condition (brown bars) or with seaweed extract + chitosan (green bars) both inoculated with *Zymoseptoria tritici (Zt)*. The dashed lines show the nutrient deficiency limits according to Bergmann (1992). **(A–C)** represent mean values of five replicates per treatment. Mean values with the same lowercase letters within each graph are not significantly different according to Tukey test (α=0.05).

No nutritional benefits were recorded in response to inoculation with MC. By contrast, SC applications increased the concentrations of Zn and Cu (**Figures 4A, C**) above the deficiency thresholds (Bergmann, 1992). The plants with nitrate fertilization exhibited particularly high Mn shoot concentrations, which were further increased by SC application (**Figure 4B**).

#### 2.1.4 Plant growth

**Figure 5:**
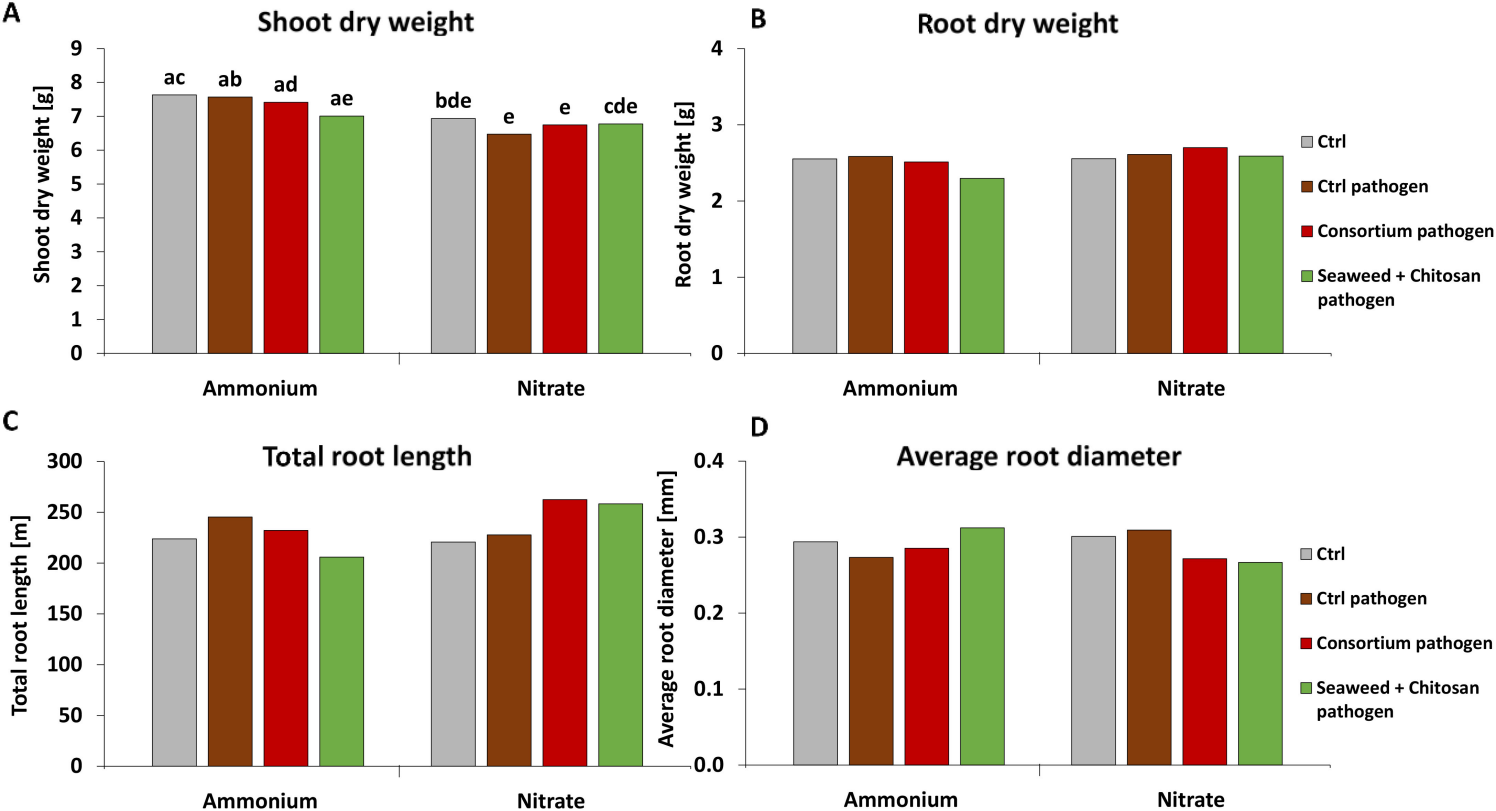
**(A)** Shoot and **(B)** root dry weight, **(C)** total root length and **(D)** average root diameter of winter wheat plants in the greenhouse 55 days after sowing (DAS) treated with ammonium sulfate or calcium nitrate under control condition with or without *Zymoseptoria tritici (Zt)* inoculum or with different biostimulants (microbial consortium, red bars; seaweed extract + chitosan, green bars) both inoculated with *Zt*. **(A–D)** represent mean values of five replicates per treatment. Mean values with at least one same or without lowercase letters within each graph are not significantly different according to Tukey test (α=0.05).

Neither the pathogen inoculation nor the application of biostimulants affected shoot and root biomass production, total root length, or root diameter (**Figure 5**). A significant decline in shoot biomass (14%) was recorded in pathogen-infected plants with NO_3_^−^ supply as compared with NH_4_^+^ fertilization (**Figure 5A**).

### 2.2 Field experiment

#### 2.2.1 Disease incidence affected by combination of biostimulants

**Figure 6:**
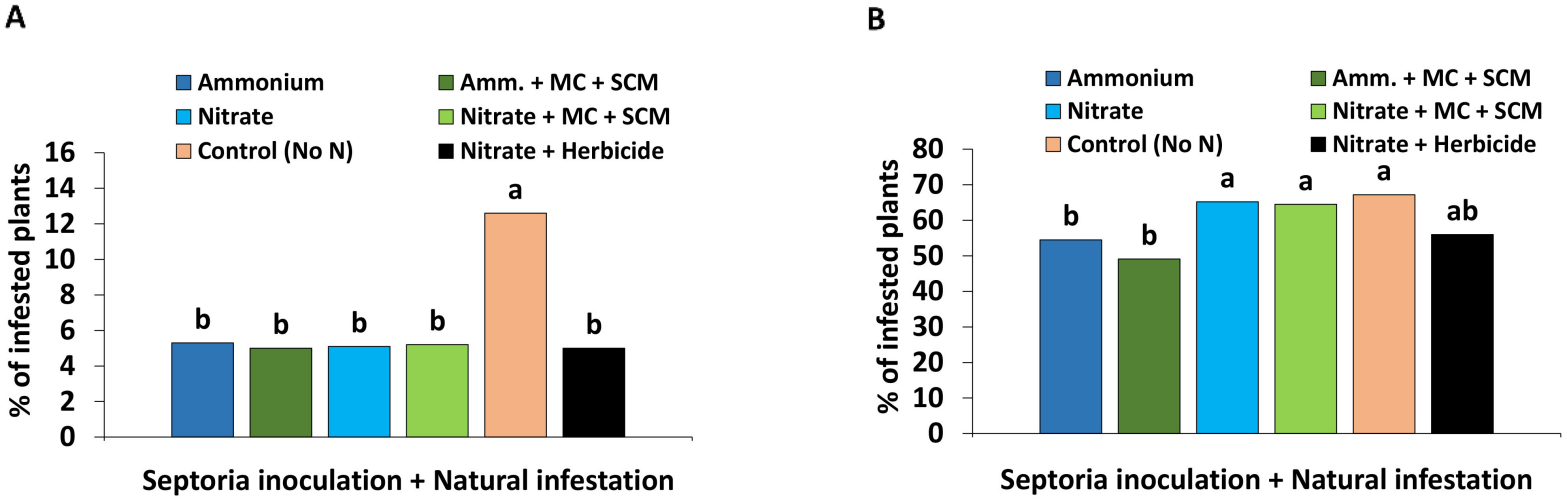
Disease incidence **(A)** 190 days after sowing (DAS) and **(B)** 259 days after sowing (DAS) [% of infested plants] of winter wheat plants in the field treated with either ammonium sulfate (Amm.) or calcium nitrate (Nitrate). In addition, both fertilizers were combined with a microbial consortium (MC) and seaweed extract + chitosan + micronutrients zinc + manganese (SCM). **(A, B)** depict a pool of *Zymoseptoria tritici (Zt)* inoculated and natural infested treatments. A negative control without nitrogen (N) (orange bars) and a positive control (black bars) both with natural infestation are included. **(A, B)** represent seaweed extract + chitosan not yet applied at the time of bonituring. **(A, B)** represent mean values of eight replicates per treatment. Mean values with at least one same lowercase letter within each graph are not significantly different according to Tukey test (α=0.05).

Due to the fact that no significant differences in *Zt* disease incidence (DI) between the *Zt*- and non-infected plants could be determined, all results with applied *Zt inoculum* and natural infestation were combined to a pool of *Zt*-inoculated and naturally infested variants in the following graphs. Independent of *Zt* inoculation, at 190 DAS (April, BBCH 30-31), first leaf blotch symptoms with a disease incidence of 5%–6% became detectable in all treatments with the exception of the control without N fertilization where DI reached already 13% (**Figure 6A**). A massive increase of DI was recorded within the next 10 weeks until 259 DAS (July, BBCH 75). Maximum DI values of approximately 65% were recorded in plants without N fertilization but similarly also under nitrate fertilization with or without application of biostimulants. Ammonium fertilization significantly reduced DI by 10% and the lowest DI values below 50% were recorded for BS-treated plants with NH_4_^+^ supply (**Figure 6B**).

#### 2.2.2 Defense-related metabolites

**Figure 7:**
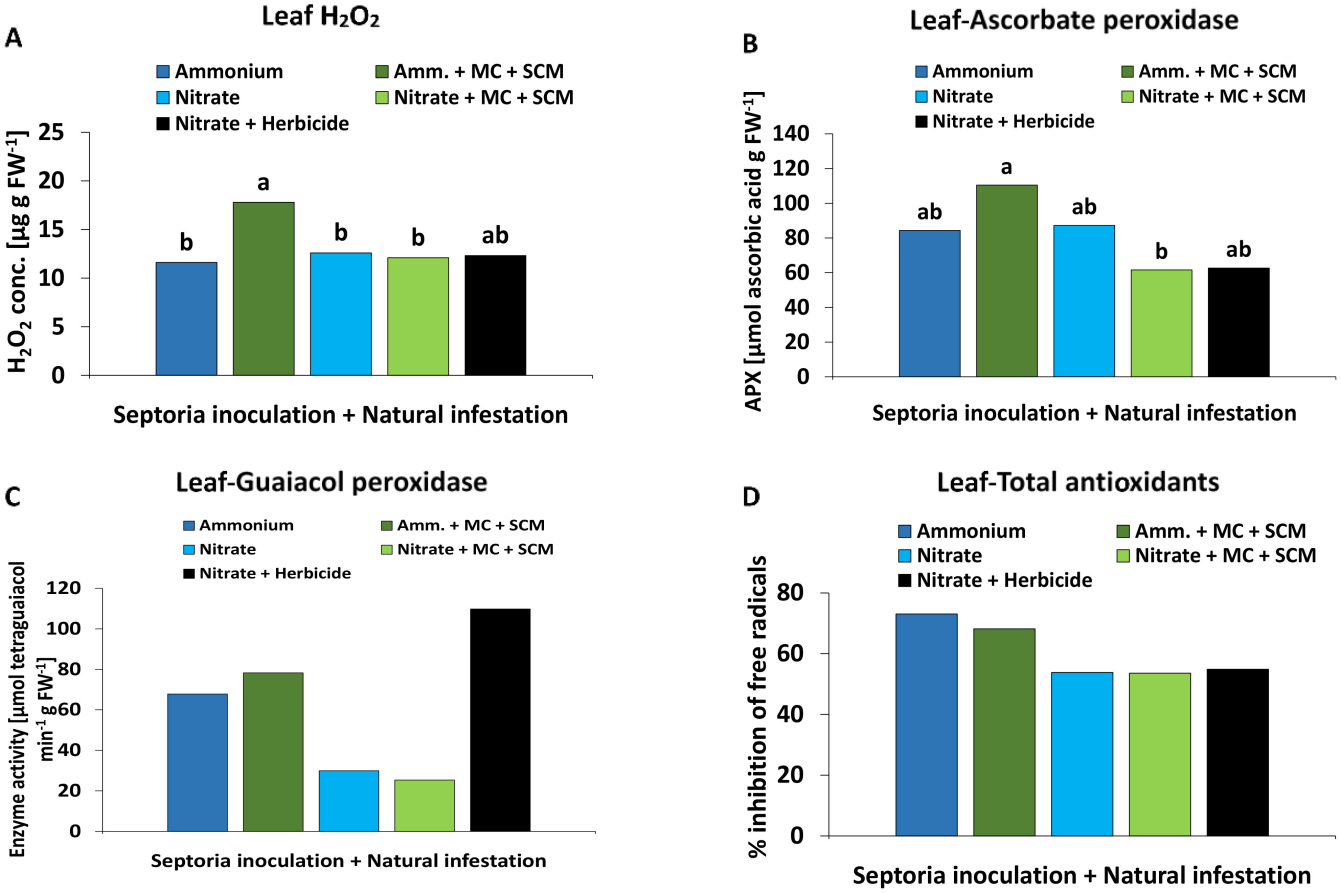
**(A)** Hydrogen peroxide (H2O2) concentration [μg g FW^-1^], **(B)** ascorbate peroxidase (APX) activity [μmol ascorbic acid g FW^-1^], **(C)** guaiacol peroxidase (GPX) activity [μmol tetraguaiacol min^-1^ g FW^-1^] and **(D)** total antioxidant potential [% inhibition of free radicals] in the leaf tissue of winter wheat plants in the field 245 days after sowing (DAS) treated with either ammonium sulfate (Amm.) or calcium nitrate (Nitrate). In addition, both fertilizers were combined with a microbial consortium (MC) and seaweed extract + chitosan + micronutrients zinc + manganese (SCM). **(A–D)** depict a pool of *Zymoseptoria tritici (Zt)* inoculated and natural infested treatments. A positive control (black bars) with natural infestation is included. **(A–D)** represent mean values of eight replicates per treatment. Mean values with at least one same or without lowercase letters within each graph are not significantly different according to Tukey test (α=0.05).

The leaf H_2_O_2_ accumulation significantly increased by approximately 50% in the BS-treated plants as compared with the remaining treatments associated also with the highest APX activity (**Figures 7A, B**). Compared with plants supplied with nitrate fertilization, ammonium fertilization tended to increase also GPX activity and accumulation of total antioxidants (**Figures 7C, D**).

#### 2.2.3 Mineral nutritional status

**Figure 8:**
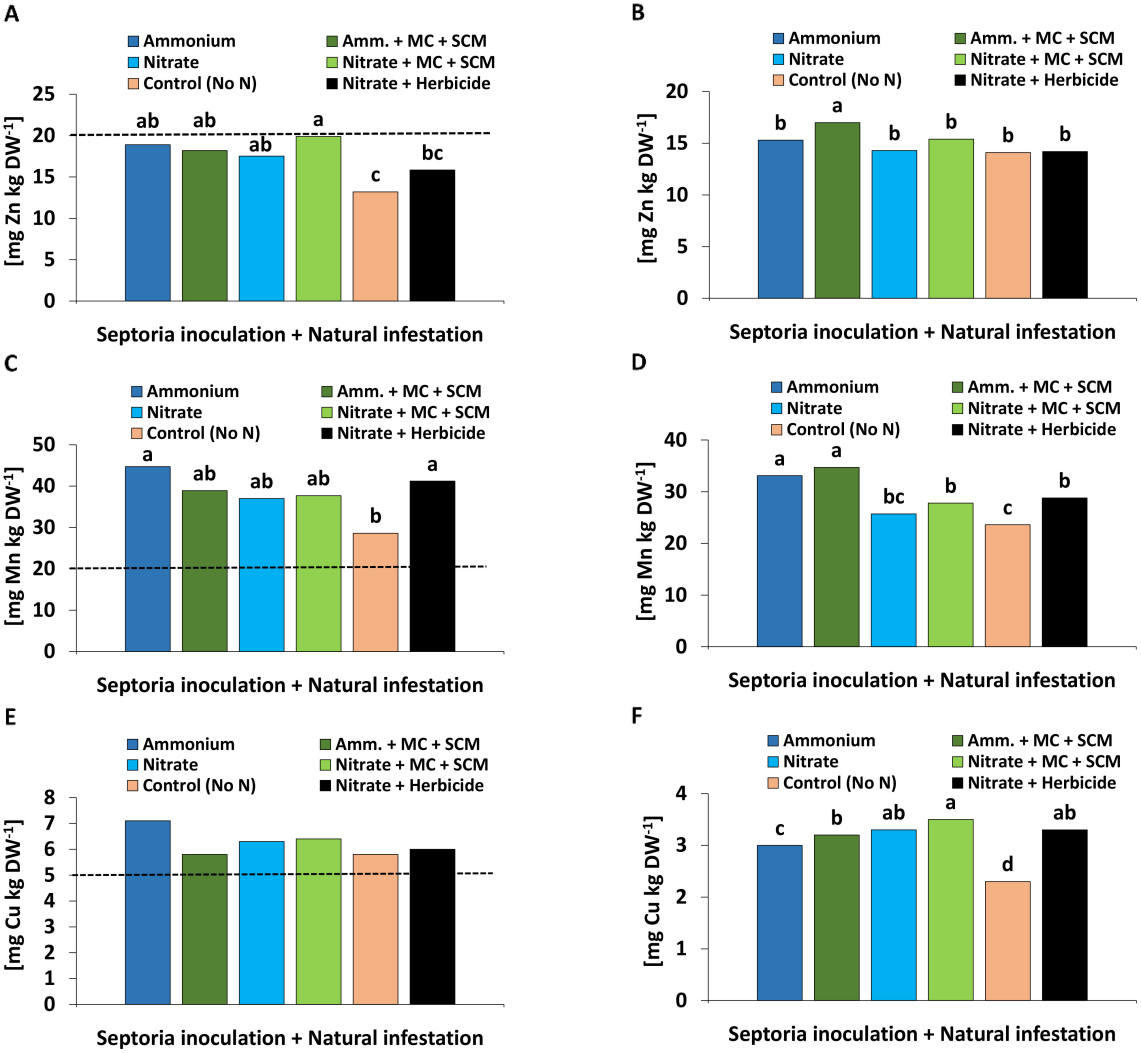
**(A)** Zinc (Zn), **(C)** manganese (Mn), **(E)** copper (Cu) concentration [mg kg DW^-1^] in the leaf tissue 209 days after sowing (DAS) and **(B)** Zn, **(D)** Mn, **(F)** Cu concentration [mg kg DW^-1^] in the grain 280 days after sowing (DAS) of winter wheat plants in the field treated with either ammonium sulfate (Amm.) or calcium nitrate (Nitrate). In addition, both fertilizers were combined with a microbial consortium (MC) and seaweed extract + chitosan + micronutrients zinc + manganese (SCM). A-F depict a pool of *Zymoseptoria tritici (Zt)* inoculated and natural infested treatments. A negative control without nitrogen (N) (orange bars) and a positive control (black bars) both with natural infestation are included. **(A, C, E)** represent seaweed extract + chitosan not yet applied at the time of bonituring. The dashed lines show the nutrient deficiency limits according to Bergmann and Neubert (1976). **(A–F)** represent mean values of eight replicates per treatment. Mean values with at least one same or without lowercase letters within each graph are not significantly different according to Tukey test (α=0.05).

With the exception of Zn, the mineral nutritional status was sufficient for all investigated nutrients (N, P, K, S, Ca, Mg, Fe, Mn, Cu) in all plants which received N fertilization. However, negative controls without N supply showed multiple nutrient deficiencies with respect to N, K, Ca, Mg, and Zn but also decreased leaf concentrations of the remaining nutrients except Cu. Compared with NO_3_^−^ fertilization, NH_4_^+^ fertilization decreased the leaf concentrations of N, K, Mg, and Ca (summarized in **Supplementary Figure 5**). The only recorded BS effect was reflected by a significantly increased Zn concentration in grains in the ammonium-treated variant. Additionally, NH_4_^+^ fertilization significantly increased grain concentrations of Mn (**Figure 8**).

#### 2.2.4 Grain yield, grain protein, and root growth characteristics

**Figure 9:**
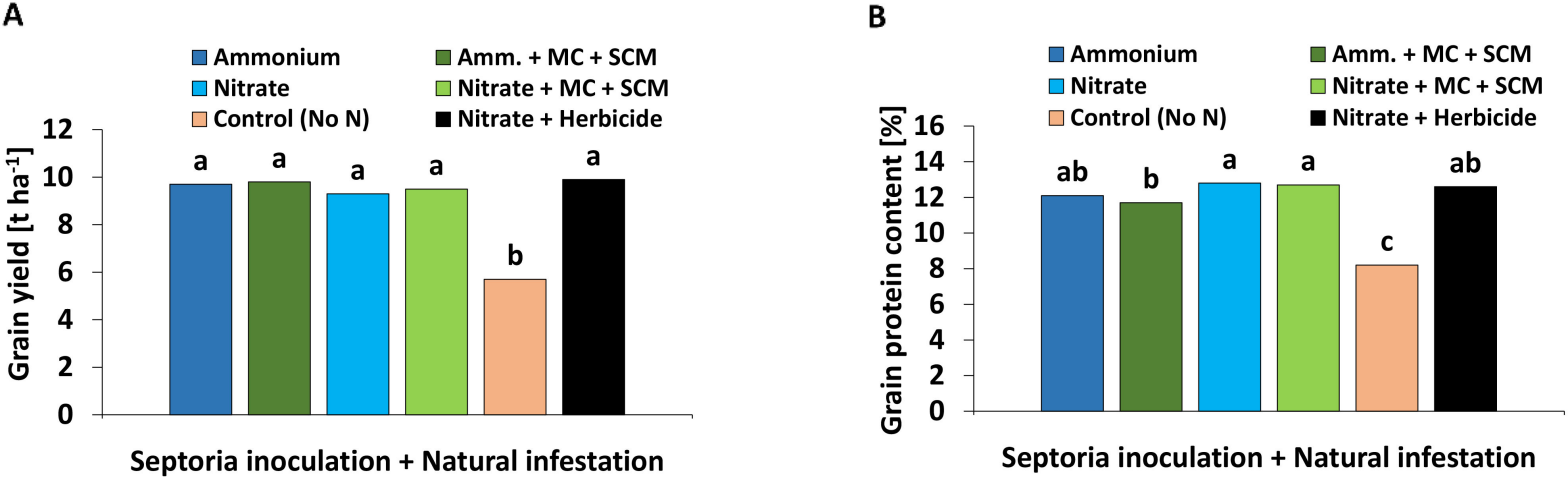
**(A)** Total grain yield [t ha^-1^] and **(B)** grain protein content [%] 280 days after sowing (DAS) of winter wheat plants in the field treated with either ammonium sulfate (Amm.) or calcium nitrate (Nitrate). In addition, both fertilizers were combined with a microbial consortium (MC) and seaweed extract + chitosan + micronutrients zinc + manganese (SCM). **(A, B)** depict a pool of *Zymoseptoria tritici (Zt)* inoculated and natural infested treatments. A negative control without nitrogen (N) (orange bars) and a positive control (black bars) both with natural infestation are included. **(A, B)** represent mean values of eight replicates per treatment. Mean values with at least one same lowercase letter within each graph are not significantly different according to Tukey test (α=0.05).

A high grain yield with an average of 9.2 t ha^−1^ was achieved in all treatments except the negative control without N supply, which showed a 40% reduction (**Figure 9A**). In all treatments receiving N fertilization, approximately 12% grain protein content was recorded with the lowest values in BS-treated plants under NH_4_^+^ fertilization. In the negative control without N fertilization, the grain protein content reached only 8% (**Figure 9B**).

For the characterization of root morphological characteristics, root length in different root diameter classes was investigated from excavated root systems. For all treatments, the proportion of fine roots (0 - 0.2 mm diameter) was very similar and comprised between 30% and 40% of the total root length (**Supplementary Figure 6**).

## 3 Discussion

### 3.1 Disease spread affected by form and amount of N supply

In the greenhouse experiment, disease spreading showed a biphasic pattern, starting with a slow increase of approximately 25% during 14 DAI–28 DAI followed by a stronger spreading of approximately 50% during the next 12 days (**Figures 1A, B**). This may be attributed to unfavorable conditions with respect to temperature (25°C–36°C) and relative humidity (<50%) during the initial phase of pathogen infection (Eyal et al., 1987; Shaw, 1991; Fones et al., 2017). Accordingly, disease spread subsequently increased with declining temperature (<25°C) and increasing relative humidity (>50%) (**Figures 1C, D**), finally reaching a moderate disease severity (DS) of 15% independent of the N form supply (NO_3_^−^ vs NH_4_^+^ during the first 8 weeks of plant development). 

Similarly also under field conditions, a low disease incidence (DI) of 5%–6%, which was independent of the applied N form (**Figure 6A**), was observed during early growth of winter wheat in spring until BBCH 30-31, associated with generally suboptimal temperatures of <15°C for *Zt* infection during this time period (Eyal et al., 1987). During the next 10 weeks, a steep DI increase (**Figure 6B**) coincided with conductive conditions, characterized by increasing temperatures, relative humidity values of 60%–90%, and precipitation above the long-term average (164%–168%) in April and June (**Supplementary Figure 7**). However, in later stages of plant development (BBCH 75), a clear N form effect was detectable with a maximum DI of 65% and the highest leaf N concentrations in plants with NO_3_^−^ fertilization. By contrast, a significantly lower DI (55%) and a lower leaf N status were recorded in plants with NH_4_^+^ supply (**Figure 6B**; **Supplementary Figure 5A**). High levels of N fertilization can improve the N-nutritional status and promote plant growth, but at the expense of reduced formation of lignin and waxy cuticles acting as physical barriers for pathogen penetration (Sun et al., 2020). Accordingly, also Simón et al. (2003) and Harrat and Bouznad (2014) reported increased disease spread for *Zt* in wheat, associated with increased N supply. In our experiment, the lower N status of plants supplied with stabilized NH_4_^+^ fertilization may be related to reduced N availability due to stronger adsorption and lower mobility of NH_4_^+^ in soils compared to NO_3_^−^ fertilizers (Marschner and Rengel, 2023). Accordingly, a stimulation of oxidative stress defense, lignification, and accumulation of epicuticular waxes under NH_4_^+^ fertilization was reported by Wang et al. (2010) and Blanke et al. (1996).

Increased DI values were similarly recorded also in wheat plants without N fertilization both during early growth (BBCH 30-31, **Figure 6A**) and in later stages of plant development (BBCH 75, **Figure 6B**). A massive decline in grain yield (**Figure 9A**) associated with leaf concentrations of N, K, Mg, Ca, and Zn below the reported deficiency thresholds (Bergmann, 1992) suggests that the respective plants were obviously affected by multiple nutrient deficiencies, weakening the expression of defense responses against pathogens.

### 3.2 Disease spread affected by microbial and non-microbial biostimulants

A microbial PGPM consortium (MC) derived from a combination of bacterial and fungal PGPM strains (*Pseudomonas*, *Bacillus*, *Trichoderma*) and a non-microbial BS combination product (SC) based on *Ascophyllum nodosum* seaweed extract, chitosan, and stress-protective micronutrients (Zn, Mn, Cu) were used for the experiments. The selection was based on literature reports suggesting synergistic or complementary benefits of BS combinations to protect plants against various biotic and abiotic stress factors (Zaim et al., 2018; Bradáčová et al., 2019; Gunupuru et al., 2019; Karuppiah et al., 2019; Moradtalab et al., 2020) to cover a wider range of environmental conditions.

In the greenhouse experiment, protective effects against *Zt* leaf blotch were recorded for both MC and SC treatments (**Figure 2**). In contrast to the untreated controls (see. 4.1), disease severity was differentially affected by the form of N supply in MC- and SC-treated plants. While the MC formulation was more effective in plants with NH_4_^+^ supply, the non-microbial SC combination responded faster compared with MC and showed stronger suppressive effects under NO_3_^−^ fertilization (**Figure 2**).

#### 3.2.1 Oxidative burst

Similar to DS, also physiological stress defense responses to BS applications were differentially influenced depending on the form of N supply. Ammonium-fertilized plants specifically responded to MC application with a selective increase in leaf H_2_O_2_ accumulation by almost 50%. A similar trend was recorded after SC application in plants with NO_3_^−^ supply (**Figure 3**). This may reflect the locally increased ROS (H_2_O_2_) production of pathogen-infected tissues (oxidative burst), frequently recorded as a first defense line against invading pathogens (Choudhary et al., 2017). Accordingly, the capacity for H_2_O_2_ production during the oxidative burst seems to be one factor determining *Zt* resistance in wheat genotypes (Shetty et al., 2003). Interestingly, after application of PGPMs or non-microbial biostimulants, the opposite response, characterized by improved ROS detoxification and declining tissue concentrations of H_2_O_2_, is frequently reported in plants affected by abiotic stress (Orzali et al., 2017; EL Mehdi EL Boukhari et al., 2020). However, in the presence of pathogens, also defense priming via increased PGPM-induced H_2_O_2_ production (mediated, e.g., by bacterial surfactants), has been described (Zhu et al., 2022). This scenario applies similarly also for the SC components chitosan (Vasil’ev et al., 2009) and *Ascophyllum nodosum* seaweed extract (Cook et al., 2018), suggesting selective effects of the investigated microbial and non-microbial BSs, depending on biotic vs abiotic stress factors.

#### 3.2.2 Detoxification of reactive oxygen species

In parallel with the stimulation of H_2_O_2_ accumulation, MC inoculation also increased the activity of APX mediating the enzymatic degradation of H_2_O_2_ (Caverzan et al., 2012) preferentially in wheat plants with NH_4_^+^ fertilization (**Figure 3B**). This effect may protect non-infected tissues located close to the infection sites from oxidative stress associated with the oxidative burst. Systemic induction of APX and other enzymes involved in ROS detoxification is a well-known response not only to inoculation with PGPM strains of *Pseudomonas*, *Bacillus*, or *Trichoderma* (reviewed by Kumudini and Patil, 2021) but also to treatments with non-microbial BSs such as seaweed extracts (EL Mehdi EL Boukhari et al., 2020) or chitosan (Orzali et al., 2017). In contrast to MC-treated plants, no comparable increase in APX activity was recorded after SC application. However, under NO_3_^−^ fertilization, the activity of GPX, known to play a central role in pathogen defense (Prakasha and Umesha, 2016), was increased in SC-treated plants. Similar to APX, also GPX activity might be expected to reduce the level of ROS by metabolizing H_2_O_2_. However, GPX is also capable of catalyzing various oxidase reactions leading to H_2_O_2_ generation and is involved in lignification, biosynthesis of ethylene, wound healing, and polysaccharide cross-linking (Sharma et al., 2012).

#### 3.2.3 Micronutrient status

SC application increased the plant micronutrient status, thereby reducing Zn and Cu deficiencies (Bergmann, 1992) of the investigated plants (**Figure 4**). However, this effect was not detected in response to MC inoculation (**Supplementary Figure 8**). Deficiency of Zn and Cu was likely related to the neutral soil pH promoting the fixation of these micronutrients (Marschner, 1995). Supplementation by foliar micronutrient supply via the SC mixture/treatment obviously provided these micronutrients as important cofactors for enzymatic ROS detoxification and the GPX-mediated reactions described above (Datnoff et al., 2007).

An extraordinarily high plant-Mn status, far above the reported deficiency threshold (Bergmann, 1992), was recorded in the pot experiment, which was even further increased by SC application under NO_3_^−^ fertilization (**Figure 4B**). In plants with NH_4_^+^ fertilization, this increase was likely counteracted by cation competition between uptake of NH_4_^+^ and Mn^2+^ (Marschner, 1995). The high Mn status may reflect an exceptionally high Mn availability in the respective soil, which was probably caused by long-time exposure to high greenhouse temperatures of 25°C–35°C (**Figure 1C**) reported to increase soil Mn availability (Reid and Racz, 1985). This may be mediated by stimulation of reductive processes such as increased respiratory oxygen consumption and modifications of microbial communities involved in Mn mobilization at higher soil temperatures especially when there is too much water and/or soil substrate compaction in the pot (Sparrow and Uren, 1987). A protective effect of Mn nutrition on controlling root or foliar diseases of plants (e.g., powdery mildew, downy mildew, take-al) is well documented (reviewed by Datnoff et al., 2007). Accordingly, also Eskandari et al. (2018) and Eskandari et al. (2020) reported improved resistance of cucumber to powdery mildew and anthracnose after foliar application of Mn, reaching similar Mn leaf concentrations >100 mg kg^−1^ DM as recorded in SC-treated plants in our study (**Figure 4**). The protective effects were attributed to improved lignification associated with increased activities of guaiacol peroxidase and phenol oxidase and increased callose production as mechanical barriers against fungal infection (Eskandari et al., 2018, 2020).

Taken together, the results suggest complementary protective effects of the MC and SC formulations against *Zt* leaf blotch, influenced by different forms of N fertilization. The defense responses, systemically induced by the MC formulation under NH_4_^+^ supply may be related to beneficial effects of NH_4_^+^ fertilizers on the establishment of plant microbial interactions. Therefore, PGPM inoculants, based on strains of *Pseudomonas*, *Bacillus*, and *Trichoderma* may promote pathogen suppression, e.g., via improved root colonization, increased auxin production or proliferation of root hairs as potential infection sites (Bradáčová et al., 2019; Mpanga et al., 2019; Moradtalab et al., 2020). The SC formulation additionally provides a source of micronutrients (Zn, Mn, Cu) with essential functions as cofactors for various physiological defense responses to pathogen infection. This is particularly important under conditions of limited micronutrient solubility at neutral to alkaline soil pH, further promoted by root-induced rhizosphere alkalization induced by NO_3_^−^ fertilization (Marschner, 1995).

#### 3.2.4 Field experiment

Under field conditions, a combined application of MC and SC with the addition of micronutrients zinc + manganese (MC-SCM application) was performed to exploit the potential benefits arising from complementary effects detected in the greenhouse experiment. At 259 DAS (BBCH 75), the highest DI of 65% was recorded under NO_3_^−^ fertilization without any benefits by MC-SCM application (**Figure 6**). By contrast, MC-SCM-treated plants supplied with NH_4_^+^ fertilization showed the lowest DI (<50%) associated with increased H_2_O_2_ accumulation and APX activity in the leaf tissue (**Figure 7**), similar to the MC responses in the greenhouse experiment. However, compared with NO_3_^−^ fertilization, the DI significantly declined to 55% even in untreated controls with NH_4_^+^ supply, demonstrating only a small additional non-significant effect of MC-SCM (**Figure 6**), at least during the investigated later stages of plant development. This applied similarly also for an improved micronutrient status (Zn, Mn, Cu) detectable in the leaf tissue and finally also in the grains (**Figure 8**).

Similarly, also in the greenhouse experiment, the protective effects of the MC/SC inoculants declined with increasing age of the plants and increasing DS (**Figure 2**). This may indicate that the applied BS products mainly promoted the early stages of pathogen defense. It may also be speculated that this reflects a downregulation of plant defense reactions induced by fungal effector proteins, characteristic for many biotrophic and hemibiotrophic pathogens including *Zt* (Yang et al., 2013; Brennan et al., 2019).

### 3.3 Plant growth and grain yield

Despite mitigation effects on DI and DS recorded in the experiments, plant growth indicators such as shoot and root biomass, root length, root diameter (**Figure 5**; **Supplementary Figure 6**), and also final grain yield (**Figure 9**) remained largely unaffected, both by pathogen inoculation or application of the microbial and non-microbial BSs. High grain yields of 9 t ha^−1^–10 t ha^−1^ reaching baking quality with grain protein contents around 12% in all treatments supplied with N fertilizers even in the presence of high DI values (50%–65%) may reflect a certain inherent disease tolerance of the investigated wheat cultivar (Asory). Asory is claimed to be a variety of medium-high resistance against *Zt* with a rating of 7.5 (Danko Saatzucht Deutschland GmbH, 2024). The high grain yields, far above the 2022 average of 7.5 t ha^−1^ in Baden-Württemberg (Statistisches Landesamt Baden-Württemberg, 2023), may indicate that there was no relevant impact of stress factors other than the *Zt*-related pathogen pressure. This could also be a reason for the lack of expression of relevant effects on plant growth in response to application of the investigated microbial and non-microbial BS products, of which benefits have been frequently proven under abiotic stress conditions (Bradáčová et al., 2016; Mpanga et al., 2019; Moradtalab et al., 2020; Rasul et al., 2021).

### 3.4 Concluding remarks

The BS-assisted fertilization strategies investigated in this study could not fully prevent but clearly slowed down *Zt*-induced disease spread, depending on the stages of plant development and the form of N fertilization. The applied BS products promoted early defense responses to pathogen attack with preferences for the microbial MC formulation if combined with NH_4_^+^ fertilization and the non-microbial SC formulation with NO_3_^−^ supply. Benefits of NH_4_^+^-dominated N fertilization correlated with an improved micronutrient status but were detectable also in later stages of plant development under field conditions. Thus, the combined application of MC and SC with the addition of micronutrients zinc + manganese (MC-SCM application) reduced the pathogen pressure with NH_4_^+^ fertilization in the field experiment by inducing increased H_2_O_2_ and APX activities. Furthermore, plant growth remained largely unaffected, under both greenhouse and field conditions. It remains to be investigated to which extent these effects can be used to replace fungicide applications during the respective time periods as part of a strategy for integrated pest management. Additional benefits may arise from protective effects reported for the applied BS products against abiotic stress factors.

## Data Availability

The raw data supporting the conclusions of this article will be made available by the authors, without undue reservation.
