# Supplementary material for: Protective potential of selected microbial and non-microbial biostimulants against Zymoseptoria tritici leaf blotch in winter wheat as affected by the form of N supply
Source: Front Plant Sci. 2024 Sep 27;15:1407585. doi: 10.3389/fpls.2024.1407585 (PMC11467867; doi:10.3389/fpls.2024.1407585)
Supplement: Supplementary file 1 [file DataSheet1.pdf]

Supplementary Material

Göbel M.*

*** Correspondence:** Göbel Markus: markus.goebel@uni-hohenheim.de

Supplementary Figures and Tables:

Supplementary Table 1 in section 1.1.1: Soil properties of "Filderlehm" (2015) for the pot experiment.

**Soil properties**	**Filderlehm (2015)**
pH ($CaCl_2$)	6.97
P_{CAL} [mg kg^{-1} soil DW]	83.35
K_{CAL} [mg kg^{-1} soil DW]	157.74
Mg $CaCl_2$ [mg kg^{-1} soil DW]	218
N (elemental analysis) [mg kg^{-1} soil DW]	16.68
sand (63 - 2000 µm) [%]	2.61
silt (2 - 63 µm) [%]	67.83
clay (< 2 µm) [%]	29.56
C total (elemental analysis) [%]	1.40
organic C (elemental analysis) [%]	1.35

main-plot 1 (pots with pathogen, variants 3-10)
main-plot 2 (pots without pathogen, variants 1-2)
main-plot 3 (pots without pathogen, variants 1-2)
main-plot 4 (pots with pathogen, variants 3-10)

row/split	1	2	3	4	5	6	7	8	9	10	
1	48	44	38	13	31	25	27	19	6	1	table 1
2	17	40	42	33	23	15	46	29	4	8	
3	5	10	22	11	34	50	37	20	26	43	table 2
4	9	3	12	39	45	16	49	24	32	28	
5	7	2	41	21	47	30	18	35	14	36	
row/split	10	9	8	7	6	5	4	3	2	1	

Supplementary Figure 1 in section 1.1.1: Split-plot design with 50 pots of the pot experiment in the greenhouse.

Supplementary Table 2 in section 1.1.1: Different variants used in the pot experiment.

Variant number	Pot numbers	N-form	Variant name
1	1-5	NH_4^+	NH_4^+ Control without pathogen
2	6-10	NO_3^-	NO_3^- Control without pathogen
3	11-15	NH_4^+	NH_4^+ Control pathogen
4	16-20	NH_4^+	NH_4^+ Consortium pathogen
5	21-25	NH_4^+	NH_4^+ Seaweed + Chitosan pathogen
6	26-30	NH_4^+	NH_4^+ Si pathogen
7	31-35	NO_3^-	NO_3^- Control pathogen
8	36-40	NO_3^-	NO_3^- Consortium pathogen
9	41-45	NO_3^-	NO_3^- Seaweed + Chitosan pathogen
10	46-50	NO_3^-	NO_3^- Si pathogen

Supplementary Table 3 in section 1.1.2: Total amount of nutrients supplied to the plants through chitosan in the pot experiment.

**Nutrient**	**Applied amount [mg pot^{-1}]**
Copper (Cu)	0.133
Manganese (Mn)	0.266
Molybdenum (Mo)	0.003
Zinc (Zn)	0.209
total Nitrogen (N)	0.665
ammoniacal Nitrogen (NH_4-N)	0.052
carbamide Nitrogen (NH_2-N)	0.614
Potassium (K)	1.105
Sulfur (S)	0.319
Chloride (Cl)	0.009

Supplementary Table 4 in section 1.2.1: Soil analyses of "Heidfeldhof soil" for the field experiment.

**Elements**	**Amount**	**Extraction method**
C [%]	1.44	EA
N [%]	0.15	EA
K [mg kg^{-1} soil DW]	3026.65	ICP-OES KW
S [mg kg^{-1} soil DW]	211.75	ICP-OES KW
Ca [mg kg^{-1} soil DW]	3922.05	ICP-OES KW
Mg [mg kg^{-1} soil DW]	2788.42	ICP-OES KW
Zn [mg kg^{-1} soil DW]	49.28	ICP-OES KW
Mn [mg kg^{-1} soil DW]	1016.99	ICP-OES KW
Fe [mg kg^{-1} soil DW]	25661.07	ICP-OES KW
Cu [mg kg^{-1} soil DW]	16.23	ICP-OES KW
K_{CAL} [mg kg^{-1} soil DW]	290.75	ICP-OES_CAL
P_{CAL} [mg kg^{-1} soil DW]	74.11	ICP-OES_CAL
pH value	6.95	$CaCl_2$

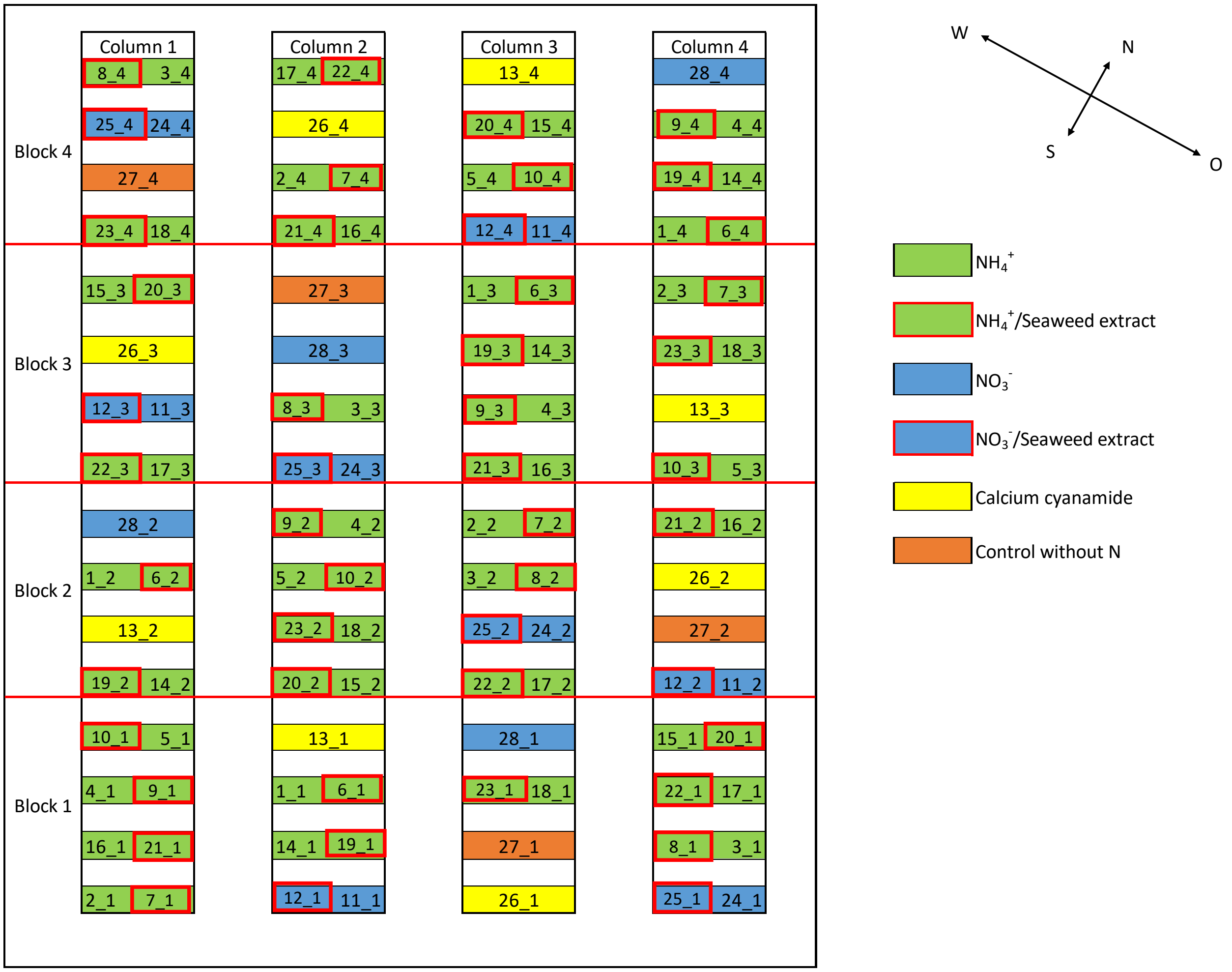

Supplementary Figure 2 in section 1.2.1: Cultivation design with 112 plots in the field experiment (row-column design with latinization in columns and blocks).

Supplementary Table 5 in section 1.2.1: Different variants used in the field experiment.

Plot number	Variant no.	Abbreviation	Pathogen	N Fertilizer	Biostimulants
1_1 - 1_4	1	Septoria/Ammonium	Septoria sp.	stab. NH4+	untreated
2_1 - 2_4	2	Sept./Amm./Milk	Septoria sp.	stab. NH4+	Milk powder
3_1 - 3_4	3	Sept./Amm./MC	Septoria sp.	stab. NH4+	Milk powder + Microbial consortium
4_1 - 4_4	4	Sept./Amm./MC/M	Septoria sp.	stab. NH4+	Milk powder + Microbial consortium + Micronutrients Zn & Mn
5_1 - 5_4	5	Sept./Amm./MC/Si	Septoria sp.	stab. NH4+	Milk powder + Microbial consortium + Si
6_1 - 6_4	6	Sept./Amm./SC	Septoria sp.	stab. NH4+	Seaweed extract + Chitosan
7_1 - 7_4	7	Sept./Amm./Milk/SC	Septoria sp.	stab. NH4+	Milk powder + Seaweed extract + Chitosan
8_1 - 8_4	8	Sept./Amm./MC/SC	Septoria sp.	stab. NH4+	Milk powder + Microbial consortium + Seaweed extract + Chitosan
9_1 - 9_4	9	Sept./Amm./MC/SCM	Septoria sp.	stab. NH4+	Milk powder + Microbial consortium + Micronutrients Zn & Mn + Seaweed extract + Chitosan
10_1 - 10_4	10	Sept./Amm./MC/SCSi	Septoria sp.	stab. NH4+	Milk powder + Microbial consortium + Si + Seaweed extract + Chitosan
11_1 - 11_4	11	Sept./Nitrate	Septoria sp.	NO3-	untreated
12_1 - 12_4	12	Sept./Nit./MC/SCM	Septoria sp.	NO3-	Milk powder + Microbial consortium + Micronutrients Zn & Mn + Seaweed extract + Chitosan
13_1 - 13_4	13	Sept./Calcium cyanamide	Septoria sp.	Calcium cyanamide	untreated
14_1 - 14_4	14	Natural./Ammonium	Natural infestation	stab. NH4+	untreated
15_1 - 15_4	15	Nat./Amm./Milk	Natural infestation	stab. NH4+	Milk powder
16_1 - 16_4	16	Nat./Amm./MC	Natural infestation	stab. NH4+	Milk powder + Microbial consortium
17_1 - 17_4	17	Nat./Amm./MC/M	Natural infestation	stab. NH4+	Milk powder + Microbial consortium + Micronutrients Zn & Mn
18_1 - 18_4	18	Nat./Amm./MC/Si	Natural infestation	stab. NH4+	Milk powder + Microbial consortium + Si
19_1 - 19_4	19	Nat./Amm./SC	Natural infestation	stab. NH4+	Seaweed extract + Chitosan
20_1 - 20_4	20	Nat./Amm./Milk/SC	Natural infestation	stab. NH4+	Milk powder + Seaweed extract + Chitosan
21_1 - 21_4	21	Nat./Amm./MC/SC	Natural infestation	stab. NH4+	Milk powder + Microbial consortium + Seaweed extract + Chitosan
22_1 - 22_4	22	Nat./Amm./MC/SCM	Natural infestation	stab. NH4+	Milk powder + Microbial consortium + Micronutrients Zn & Mn + Seaweed extract + Chitosan
23_1 - 23_4	23	Nat./Amm./MC/SCSi	Natural infestation	stab. NH4+	Milk powder + Microbial consortium + Si + Seaweed extract + Chitosan
24_1 - 24_4	24	Nat./Nitrate	Natural infestation	NO3-	untreated
25_1 - 25_4	25	Nat./Nit./MC/SCM	Natural infestation	NO3-	Milk powder + Microbial consortium + Micronutrients Zn & Mn + Seaweed extract + Chitosan
26_1 - 26_4	26	Nat./Calcium cyanamide	Natural infestation	Calcium cyanamide	untreated
27_1 - 27_4	27	Nat./Control	Natural infestation	Control without N	untreated
28_1 - 28_4	28	Nat./Nitrate/Herbicide	Nat. infest. with herbicides	NO3-	untreated

Supplementary Table 6 in section 1.2.2: Total amount of nutrients supplied to the plants through chitosan in the field experiment.

Nutrient	Total amount applied [g plot^{-1}]	Total amount applied [µg plant^{-1}]
Copper (Cu)	0.14	10.3
Manganese (Mn)	0.27	19.9
Molybdenum (Mo)	0.00	0.0
Zinc (Zn)	0.21	15.5
total Nitrogen (N)	0.68	50.1
ammoniacal Nitrogen (NH_4-N)	0.05	3.7
carbamide Nitrogen (NH_2-N)	0.63	46.4
Potassium (K)	1.13	83.2
Sulfur (S)	0.33	24.3
Chloride (Cl)	0.01	0.7

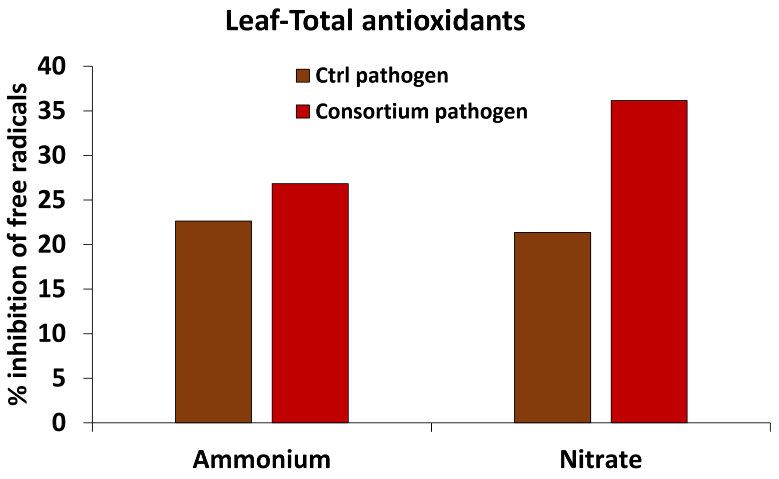

Supplementary Figure 3 in section 2.1.2: Total antioxidant potential [% inhibition of free radicals] in the leaf tissue of winter wheat plants in the greenhouse 27 days after sowing (DAS) treated with ammonium sulfate or calcium nitrate under control condition (brown bars) or with consortium (red bars) both inoculated with *Zymoseptoria tritici (Zt)*. The graphic represents mean values of three replicates in the ammonium control, two replicates in the ammonium-consortium and four replicates in nitrate control and nitrate-consortium treatments. Mean values without lowercase letters within each graph are not significantly different according to Tukey test (α=0.05).

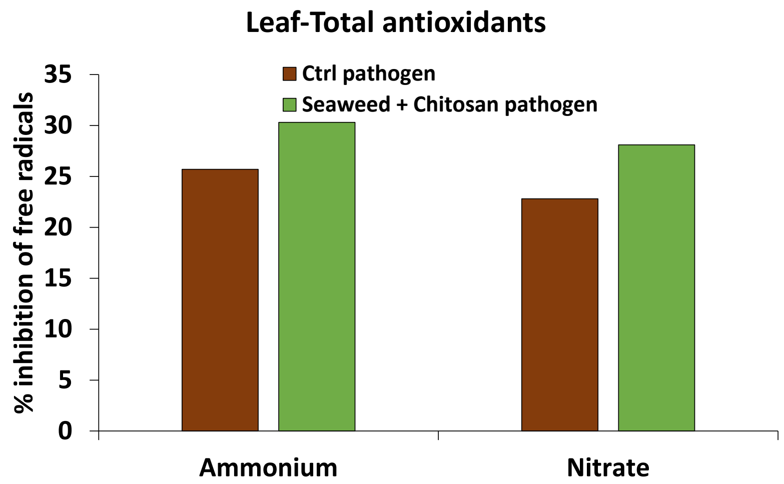

Supplementary Figure 4 in section 2.1.2: Total antioxidant potential [% inhibition of free radicals] in the leaf tissue of winter wheat plants in the greenhouse 27 days after sowing (DAS) treated with ammonium sulfate or calcium nitrate under control condition (brown bars) or with seaweed extract + chitosan (green bars) both inoculated with *Zymoseptoria tritici (Zt)*. The graphic represents mean values of three replicates in the ammonium control and four replicates in the ammonium-seaweed extract + chitosan, nitrate control and nitrate-seaweed extract + chitosan treatments. Mean values without lowercase letters within each graph are not significantly different according to Tukey test (α=0.05).

Supplementary Table 7 in section 2.1.3: Different leaf nutrient concentrations of all harvested treatments (55 DAS) of the pot experiment.

Variants	P [g kg DW^{-1}]	S [g kg DW^{-1}]	Zn [mg kg DW^{-1}]	Ca [g kg DW^{-1}]	Mg [g kg DW^{-1}]	K [g kg DW^{-1}]	Mn [mg kg DW^{-1}]
NH_4^+ Control without pathogen	3.98	3.59	24.23	3.93	1.37	32.29	84.24
NO_3^- Control without pathogen	3.58	3.11	20.79	4.50	1.47	32.50	106.89
NH_4^+ Control pathogen	4.04	3.69	24.52	3.89	1.34	31.86	84.58
NH_4^+ Consortium pathogen	4.20	3.91	25.45	4.15	1.36	32.43	78.41
NH_4^+ Seaweed + Chitosan pathogen	4.13	4.11	27.71	4.30	1.39	33.77	80.38
NH_4^+ Si pathogen	4.20	4.22	25.28	4.45	1.42	33.40	78.30
NO_3^- Control pathogen	3.66	3.66	21.72	4.75	1.51	33.95	99.14
NO_3^- Consortium pathogen	3.79	3.44	23.33	4.83	1.56	34.52	114.42
NO_3^- Seaweed + Chitosan pathogen	3.64	3.53	25.82	4.99	1.61	34.26	127.04
NO_3^- Si pathogen	3.55	3.49	23.10	4.82	1.56	35.59	110.33

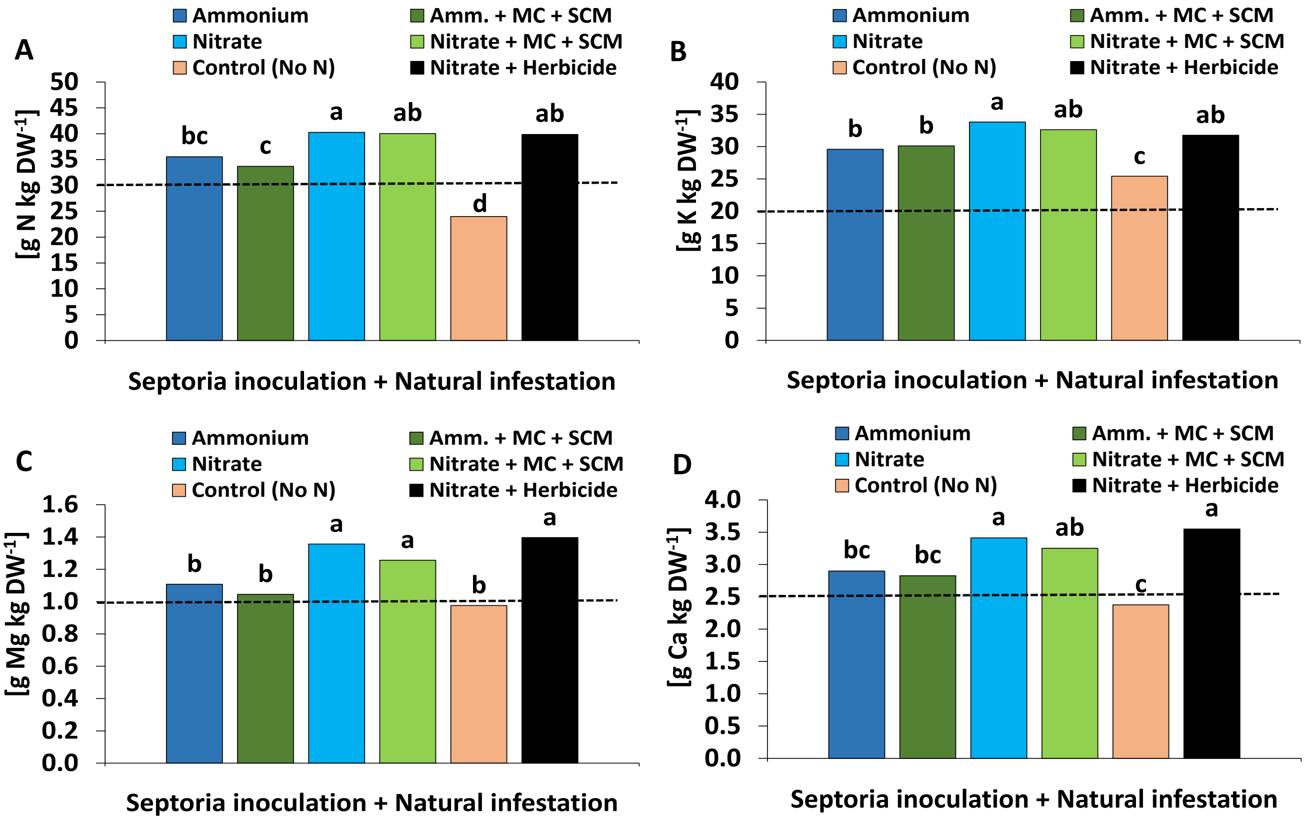

Supplementary Figure 5 in section 2.2.3: (A) Nitrogen (N), **(B)** potassium (K), **(C)** magnesium (Mg) and **(D)** calcium (Ca) concentration [g kg DW^{-1}] in the leaf tissue 209 days after sowing (DAS) of winter wheat plants in the field treated with either ammonium sulfate (Amm.) or calcium nitrate (Nitrate). In addition, both fertilizers were combined with a microbial consortium (MC) and seaweed extract + chitosan + micronutrients zinc + manganese (SCM). **A-D** depict a pool of *Zymoseptoria tritici (Zt)* inoculated and natural infested treatments. A negative control without nitrogen (N) (orange bars) and a positive control (black bars) both with natural infestation are included. The graphics represent seaweed extract + chitosan not yet applied at the time of bonituring. The dashed lines show the nutrient deficiency limits according to Bergmann (1976). **A-D** represent mean values of eight replicates per treatment. Mean values with at least one same lowercase letter within each graph are not significantly different according to Tukey test (α=0.05).

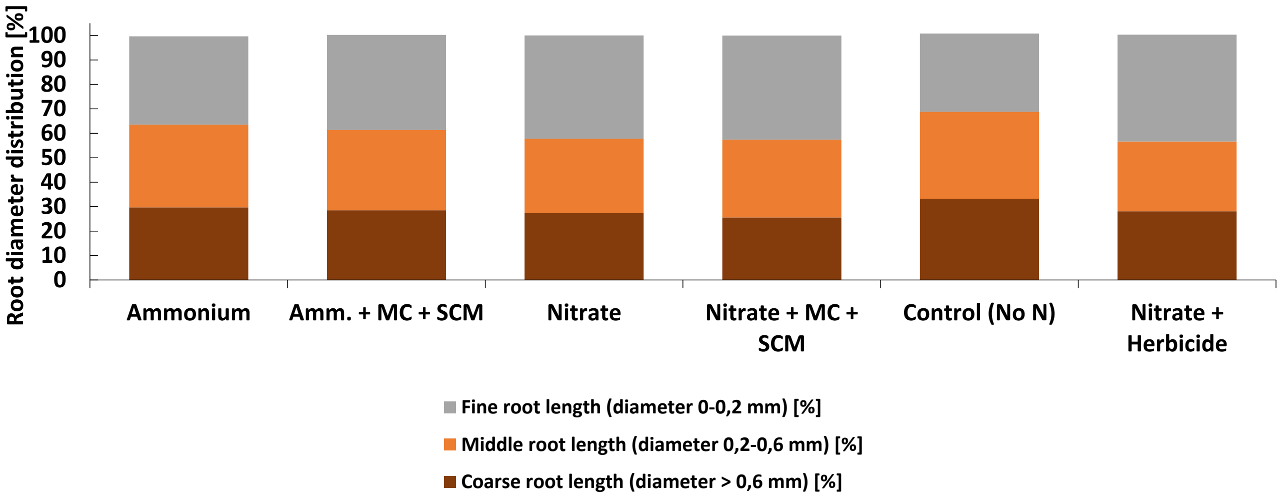

Supplementary Figure 6 in section 2.2.4: Root diameter distribution [%] 278 days after sowing (DAS) of winter wheat plants in the field treated with either ammonium sulfate (Amm.) or calcium nitrate (Nitrate). In addition, both fertilizers were combined with a microbial consortium (MC) and seaweed extract + chitosan + micronutrients zinc + manganese (SCM). The figure depicts a pool of *Zymoseptoria tritici (Zt)* inoculated and natural infested treatments. A negative control without nitrogen (N) and a positive control (Nitrate + Herbicide) both with natural infestation are included. Mean values of eight replicates per treatment are represented.

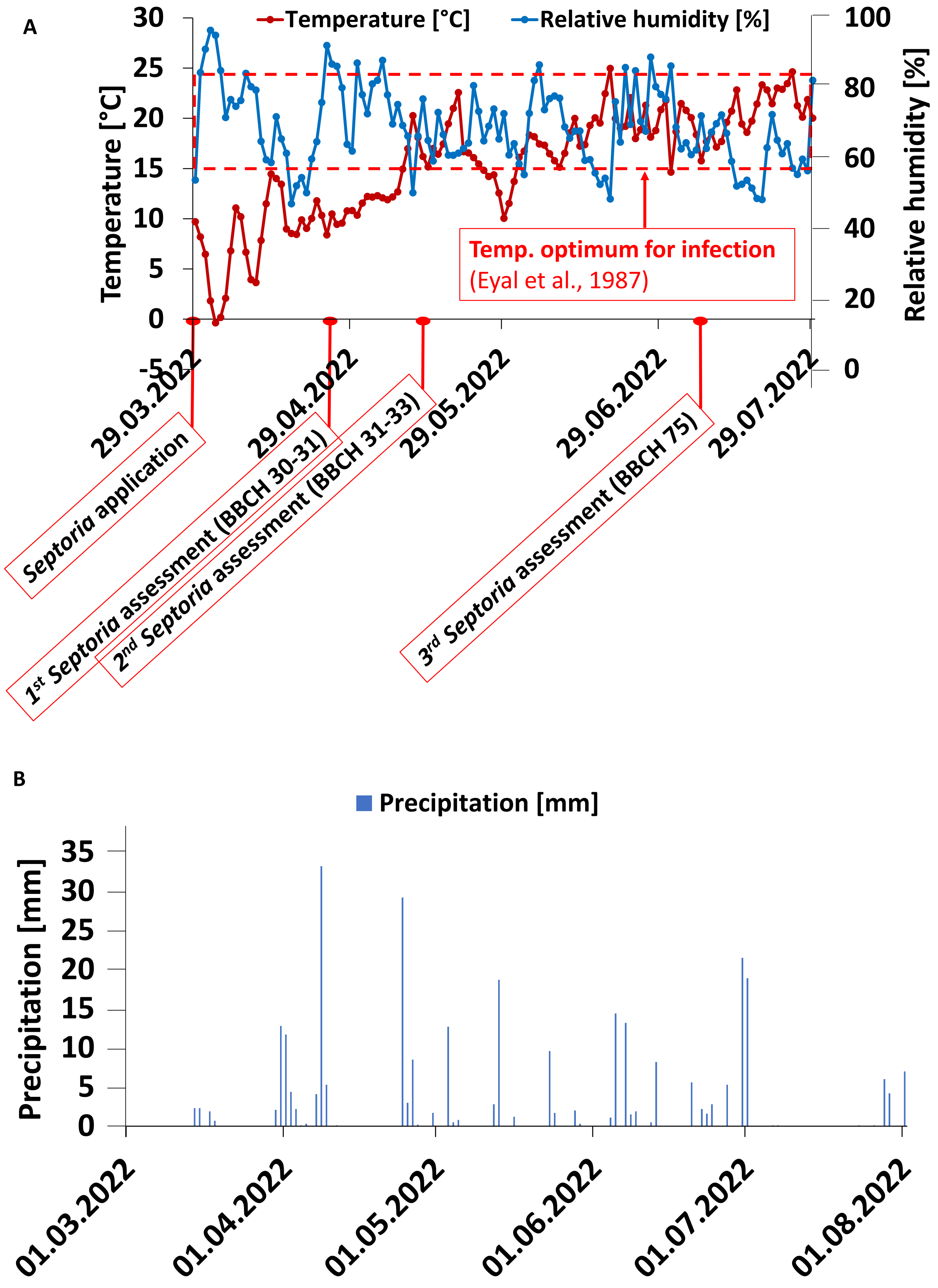
A
Temperature [°C]
Relative humidity [%]
Temp. optimum for infection
(Eyal et al., 1987)
29.03.2022
29.04.2022
29.05.2022
29.06.2022
29.07.2022
Septoria application
1st Septoria assessment (BBCH 30-31)
2nd Septoria assessment (BBCH 31-33)
3rd Septoria assessment (BBCH 75)
B
Precipitation [mm]
01.03.2022
01.04.2022
01.05.2022
01.06.2022
01.07.2022
01.08.2022

Supplementary Figure 7 in section 3.1: (A) Mean daily temperature in 20 cm height [°C] and relative humidity [%] from the day of *Zt* application on 29.03.2022 until harvest on 27.07.2022 including the *Zt* assessment days on 28.04.2022, 12.05.2022 and 06.07.2022. **(B)** Mean daily precipitation [mm] from 01.03.2022 until 01.08.2022. Data of **A, B** are from the weather station Hohenheim-Heidfeldhof (389 m).

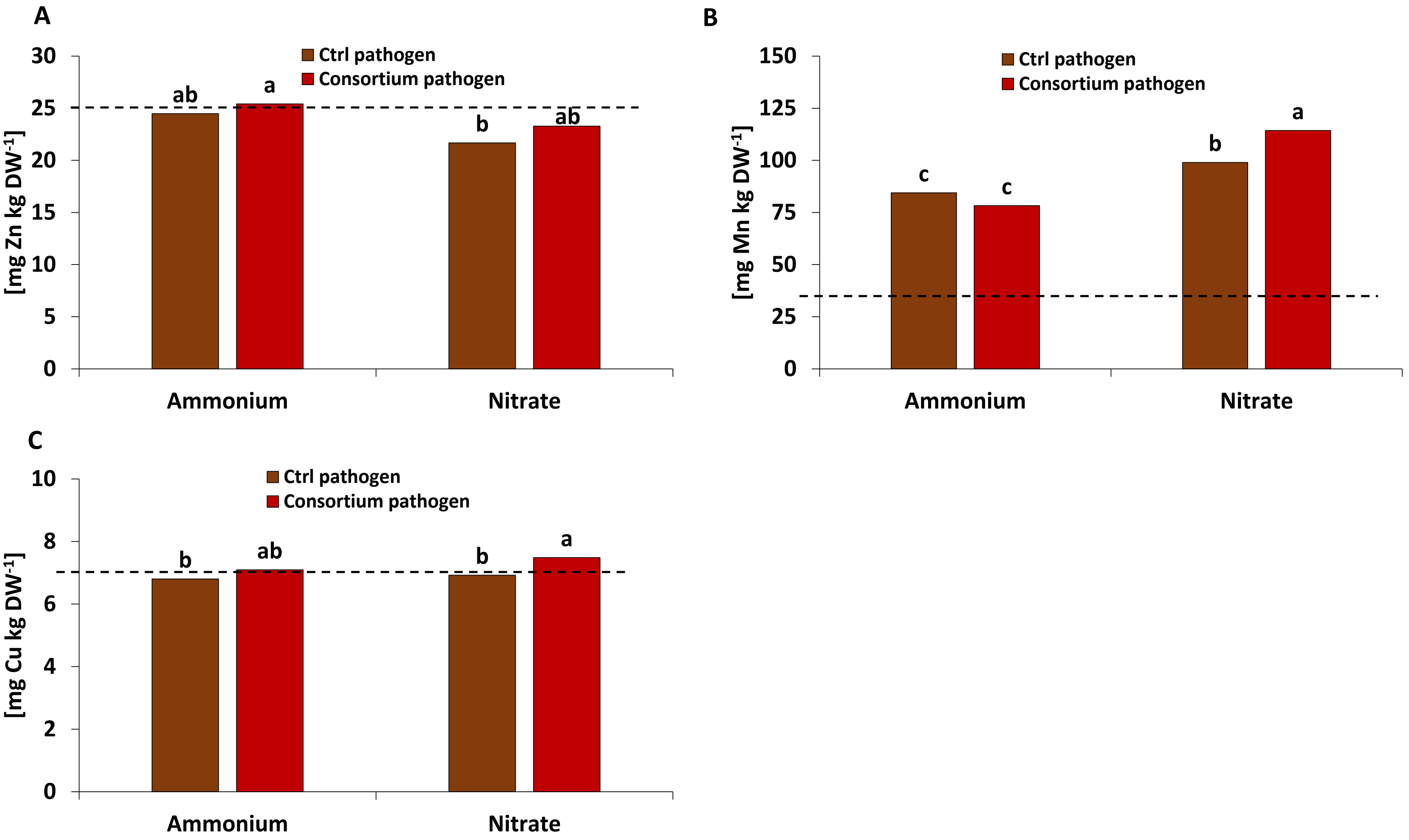

Supplementary Figure 8 in section 3.2.3: (A) Zinc (Zn), **(B)** manganese (Mn), **(C)** copper (Cu) concentration [mg kg DW^{-1}] in the shoot tissue of winter wheat plants in the greenhouse 55 days after sowing (DAS) treated with ammonium sulfate or calcium nitrate under control condition (brown bars) or with consortium (red bars) both inoculated with *Zymoseptoria tritici (Zt)*. The dashed lines show the nutrient deficiency limits according to Bergmann (1992). **A-C** represent mean values of five replicates per treatment. Mean values with at least one same lowercase letter within each graph are not significantly different according to Tukey test (α=0.05).
